# Oral Health-Related Quality of Life in Adolescents as Measured with the Child-OIDP Questionnaire: A Systematic Review

**DOI:** 10.3390/ijerph182412995

**Published:** 2021-12-09

**Authors:** María Paloma Alvarez-Azaustre, Rossana Greco, Carmen Llena

**Affiliations:** 1 Department of Dentistry, Faculty of Biomedical and Health Sciences, Universidad Europea de Valencia, Paseo de la Alameda 7, 46010 Valencia, Spain; alvarezazaustrepaloma@gmail.com; 2 Department of Stomatology, Faculty of Medicine and Dentistry, Universitat de València, c/Gascó Oliag 1, 46010 Valencia, Spain; dr.rossanagreco@gmail.com

**Keywords:** oral health, quality of life, adolescent, children, Child-OIDP, OIDP

## Abstract

Oral health-related quality of life (OHRQoL) refers to impacts of oral health on physical, psychological, functional and social aspects of individuals. Among specific measurement instruments to assess OHRQoL in adolescents, the C-OIDP (Child Oral Impact on Daily Performances) questionnaire has demonstrated validity, reliability and suitable psychometric properties. Our aim was to identify cross-sectional studies using the C-OIDP questionnaire to perform a qualitative synthesis and assessment of their methodology and results. A literature electronic search was carried out on the PubMed-Medline, Scopus, Web of Science (WoS), EMBASE, LILACS and SciELO databases, followed by a study selection process and quality assessment. OHRQoL perceived by adolescents is related to age, sex and sociodemographic factors. Eating is the most frequently affected dimension and toothache is the first cause of impact, showing a generally mild intensity and severity of impact. The impact on oral quality of life is greater in younger adolescents. Several factors such as previous caries experience, the DMFT (Decayed, Missed, Filled, Tooth) index, caries in primary teeth, canker sores, bleeding gums and malocclusion have been associated with a lower level of OHRQoL. More longitudinal studies are needed to clarify divergent results and complete our knowledge of oral impacts on quality of life.

## 1. Introduction

Oral health is an integral part of general health and wellbeing and a prominent factor influencing people’s quality of life [1]. The World Health Organization (WHO) stresses that health is a person’s right [2]. However, inequalities regarding access to health services remain an unsolved problem in many countries, which means that many people experience inequalities in healthcare and a negative impact on their health and quality of life.

Oral health-related quality of life (OHRQoL) is a multidimensional construct that includes the subjective evaluation of the state of oral health, functional and emotional well-being, expectations of and satisfaction with the dental care received, and self-esteem. It encompasses the impact of oral health on physical, psychological, functional and social aspects of individuals [3,4]. Oral health can be considered a relevant factor in the perception that individuals have of their health [5]; it exerts an influence on their self-esteem and position in life [6].

Authors such as Locker et al. [7] highlight the scarce attention that has been given to the impact that oral health has on quality of life and emphasize the need for a holistic approach in which not only clinical conditions are valued, but the individual perception of oral health is also taken into account, together with the social and psychological impact it entails.

Based on the above considerations, in the field of dentistry, several instruments have been developed to measure OHRQoL. Among the most used questionnaires, the “Oral Health Impact Profile” (OHIP) stands out, the focus of which is based on the frequency of perceived impacts [8]. It consists of 49 questions and covers seven dimensions. One of the problems that arises is its extension. To overcome this limitation, a reduced format with 14 questions was proposed, which was validated by demonstrating sufficient psychometric validity. The “Geriatric Oral Health Assessment Index” (GOHAI), aimed at assessing the impact of oral problems in the elderly population, consists of 12 questions that are included in a single dimension [9]. The “Dental Impact on Daily Living” (DIDL) was designed to study the oral impacts of the Brazilian adult population [10], consisting of 36 items belonging to five dimensions. The “Oral Impact on Daily Performances” (OIDP) questionnaire was designed to measure the frequency and severity of the impacts of oral conditions on eight activities of people’s daily life [11]. It was validated in Spain for the adult population in 2008 [12] and has been used in various socioeconomic contexts and specific populations from different settings.

A recent systematic review evaluated the knowledge about the general and psychometric characteristics of the instruments used to measure the OHRQoL. It described how all the studies that presented information on the internal consistency of their instruments reported adequate discriminant validity, and the reliability and construct validity criteria were also present in most of the studies [13]. Studies conducted in different populations and at different ages were included in this review.

To specifically measure the oral health-related quality of life of children and adolescents, specific questionnaires have been developed. To our knowledge, they are the following: Child Perception Questionnaire (CPQ11–14) [14], the Michigan OHRQoL [15], Child Oral Health Impact Profile (COHIP) [16] and the Child-Oral Impact on Daily Performances (C-OIDP) [17]. The C-OIDP is derived from the OIDP, with editorial modifications that address children’s ability in relation to their intellectual, cognitive and language development. It is based on a modified version of the WHO International Classification of Impairments, Disabilities and Handicaps and has been validated in different countries and languages, demonstrating a high validity and suitable psychometric properties [18,19,20,21].

A recent meta-analysis of the C-OIDP questionnaire found that the majority of publications reported a Cronbach’s alpha of 0.7 or higher, demonstrating appropriate internal consistency [22].

The objective of this systematic review was to identify cross-sectional descriptive studies of OHRQoL using the C-OIDP questionnaire in the 11–18-year-old school setting, carried out in the last 17 years, and to perform a qualitative synthesis and assessment of their methodologies and relevant results reported.

## 2. Materials and Methods

This systematic review followed the Preferred Reporting Items for Systematic Reviews and Meta-Analyses (PRISMA-2020) statement (Figure S1) [23]. The protocol was registered in PROSPERO, registration reference number CRD42020222392 (Figure S2).

### 2.1. Information Sources and Search Strategy

A systematic electronic literature search was carried out on the PubMed-Medline, Scopus, Web of Science (WoS), EMBASE, LILACS and SciELO databases on 4 February 2021. A further electronic search was performed on the Google Scholar bibliographic database, to find additional studies. The search strategy included the following MeSH search terms combined with Boolean Operators AND/OR: (oral health quality of life) AND (adolescents or children or scholars) AND (Child-OIDP or OIDP). No limits were imposed in terms of publication date or language. An updated search was performed on 28 July 2021, to search for additional eligible studies (Figure S3).

The reference lists from the selected studies were also reviewed manually to look for additional studies that could be eligible.

### 2.2. Eligibility Criteria

The eligibility criteria were established using the PIO question strategy, as follows: 11–18-year-old adolescents (P: Population) subjected to C-OIDP questionnaire (I: Intervention) to measure their oral health-related quality of life (O: Outcomes).

Inclusion criteria were: descriptive cross-sectional studies on adolescents aged 11–18 years old, using C-OIDP or OIDP as a measurement instrument, published in the last 17 years (2005–2021), written in English or Spanish.

Exclusion criteria were: systematic reviews, meta-analysis, literature reviews and study design other than descriptive cross-sectional studies, as well as the assessment of other age ranges and types of questionnaire (Table 1).

### 2.3. Selection Process

Once studies were identified through database searching and other sources, duplicates were removed using Mendeley reference manager software (Mendeley Desktop version 1.19.4 © 2021–2019).

After discarding repeated records, reference titles and abstracts were screened by two independent reviewers (M.P.A.-A. and R.G). Selection was focused on descriptive transversal studies in adolescent schoolchildren using C-OIDP including or not including a clinical oral exam. Descriptive cross-sectional studies using other OHRQoL questionnaires were excluded, as well as those using the C-OIDP but that were not descriptive cross-sectional studies in schoolchildren.

Criteria followed for excluding articles at this stage were: use of another type of OHRQoL questionnaire, studies aiming to measure psychometric properties (validity and feasibility) of C-OIDP, studies directed specifically at cross-cultural translation and adaptation, validation studies, studies only assessing dental caries in the concurrent oral exam, studies relating C-OIDP to specific systemic diseases, studies relating C-OIDP with only one specific oral condition and studies comparing condition-specific and generic C-OIDP questionnaires.

Once non-applicable records were excluded, full-text articles assessed for eligibility were read and analyzed and another set was found not meeting the objective of this systematic review. These were removed, rendering a final set of selected studies to be considered in the qualitative synthesis.

In case of any disagreement, a third reviewer was consulted (C.L.L.).

### 2.4. Data Collection Process and Variables

Data extraction from reports included in qualitative synthesis was performed in duplicate by two independent researchers (M.P.A.-A. and R.G.).

The following variables were analyzed: author, year, country, aim, sample selection method, sample size, age range or mean age, gender (%), type of questionnaire (generic C-OIDP or condition-specific CS-C-OIDP), questionnaire completion method and administration, type of intervention, sample’s inclusion and exclusion criteria and results (impact prevalence; mean C-OIDP score).

Data were summarized in a Microsoft Office Excel 2013 spreadsheet (Microsoft Corporation, Redmond, WA, USA) (Table 2).

### 2.5. Risk of Bias in Individual Studies and Quality Assessment

The risk of bias of each study was assessed by two independent reviewers (M.P.A.-A. and R.G.) using two complementary systems: an evidence table based on FLC 3.0 (Ficha Lectura Crítica) [24] and the STROBE checklist for cross-sectional studies (STrengthening the Reporting of OBservational studies in Epidemiology) [25] (Tables S1 and S2).

As the systematic review was focused on cross-sectional studies, and given the lack of validated methodological quality assessment scales for this specific type of research design, the quality of the studies was assessed by filling in an individual evidence table for each study, based on FLC 3.0 [24], comprising the following six areas: PIO question (Population, Intervention, Outcome) clearly settled; method description including type of study design, objectives clearly specified, setting and time where the study was carried out, description of eligibility criteria identified as inclusion and exclusion criteria, type of statistical analysis done clearly explained and rationale given; results correctly described and synthesized; proper justification of conclusions; conflicts of interest described; external validity with an assessment of the possibility to generalize the results to the general population (Table S2).

Compliance with each area was identified as: yes/no/partially/without information.

Once the “method” area was identified as yes/no/partially, the quality of the study was established as high/medium/low according to the majority of other criteria being considered as yes/no/partially [24], i.e., a study identified as “partially” in the “Method” section would be assessed as “medium quality” if the majority of other criteria were assessed as “yes/partially” (Table S2).

The STROBE checklist allowed us to identify the number of items each study complied with, providing additional information for the risk of bias and quality assessment [25].

**Table 2:** Characteristics of included studies investigating oral quality of life in adolescents using the C-OIDP questionnaire in the 11–18-year-old adolescents.

Author Year Country	Study’s Aim	Sample Selection Method Sample Size (*n*)	Age Range Sex (%)	Questionnaire (OHRQoL) Completion Mode Administration Context	Type of Intervention	Sample’s Inclusion and Exclusion Criteria	Results (Impact Prevalence; Mean C-OIDP Score)	Quality of Study (FLC 3.0/ STROBE Cross-Sectional Studies)
**Alzahrani** **et al.,** **2019** **Saudi Arabia** [26]	To examine the associations between the OHRQoL based on the Child-OIDP index and the different oral diseases among Saudi schoolchildren living in the Albaha region of Saudi Arabia.	Two-stage randomized sampling technique *n* = 349	12–15 years old Male: 100	C-OIDP Interview Three intermediate schools	Questionnaire Oral clinical examination	Inclusion: Physically and mentally fit for this study; parent’s written informed consent. Exclusion: Histories of antibiotic therapy and/or systemic diseases during the previous three months; female schoolchildren.	Impact prevalence: 75.1% Mean C-OIDP score: 2.5	Medium/18
**Bakhtiar** **et al.** **2014** **Iran** [27]	To assess the association between OHRQoL and clinical oral health measures among mid-level school children in the city of Kerman, Southeast of Iran and also, answer this question whether the status of oral health can modify OIDP index in adolescents.	Random Cluster Sample *n* = 400	11–13 years-old Male: 46.75 Female: 53.25	C-OIDP Self-completed part Interview Mid-level schools	Questionnaire Oral clinical examination	Exclusion: serious medical problem and any condition influencing on their quality-of-life and also their oral health like orthodontic treatment.	Impact prevalence: 82% Mean C-OIDP score: 10.2 C-OIDP score: 7.1	Medium/16
**Basavaraj** **et al.,** **2014** **India** [28]	To investigate whether a relationship exists between specific clinical dental measures and OHRQoL using the Child-OIDP index among children attending various schools located in Modinagar, India.	Two-stage cluster sampling technique *n* = 900	12 and 15 years old Male: 67 Female: 33 576 (64%): 12 years (385: males, 191: females) 324 (36%): 15 years (218: males, 106: females)	C-OIDP Interviewer-administered Six public and ten private middle and high schools	Questionnaire Oral clinical examination	Inclusion: 12 and 15 years old, attending various schools in Modinagar. Exclusion: Systemic diseases and on antibiotic therapy in the previous six months.	Impact prevalence: 60% Mean C-OIDP score: 2.49	High/20
**Castro et al.,** **2011** **Brazil** [29]	To assess the association between OHRQoL, measured through the Child-OIDP, and demographic characteristics, self-reported oral problems and clinical oral health measures among 11- to 12-year-old schoolchildren in the city of Rio de Janeiro, Brazil.	Probabilistic sample with complex design *n* = 571	11–12 years old Male: 38.6 Female: 61.4	C-OIDP Self-administrated part (refers to list of pathologies) Face-to-face interview part Six to seven years of public education	Questionnaire Oral clinical examination	Inclusion: Year 6 and 7 classes, 11 and 12 years old, both sexes, formally enrolled in the public educational system of the city of Rio de Janeiro, parent’s informed consent.	Impact prevalence: 88.7% Mean C-OIDP score: 7.1	Medium/16
**Do et al.,** **2020** **Vietnam** [30]	To assess the impact of oral health problems on daily activities of 12- and 15-year-old children in Can Tho.	Cluster sampling of probability proportional to size *n* = 809 *n* = 407 children of 12 years old *n* = 402 children of 15 years old	12–15 years old Sex: Not stated	C-OIDP Self-administrated part (refers to list of pathologies) Questionnaire: Interview administrated under the guidance and interpretation of the investigators Ten secondary schools (six schools in urban and four in rural areas)	Questionnaire	Inclusion: 12–15 years old, informed consent, year 6 to 9 classes.	Impact prevalence: 87–78.6% Mean C-OIDP score: 9.1–5.6	High/17
**Dumitrache et al.,** **2009** **Romania** [31]	To assess the prevalence and severity of the oral health impact on the quality of life of schoolchildren in Bucharest using the Child-OIDP index.	Random selection *n* = 413	11–13 years old Male: 47 Female: 53	C-OIDP interview administrated Six schools	Questionnaire Oral clinical examination	Inclusion: 11–13 years, randomly selected from six schools from the six-city district, parents’ and school officials’ written consent.	Impact prevalence: 57.4% Mean C-OIDP score: Not stated	Low/14
**Kumar et al.,** **2015** **India** [32]	To evaluate the psychometric properties of the Hindi version of the Child-OIDP and to estimate the oral impacts on daily performance in 12–15-year-old public and private schoolchildren. This article also aimed to determine the prevalence of dental caries in this age group.	Two-stage stratified cluster random sampling *n* = 690	12–15 years old Male: 50.724 Female: 49.28	C-OIDP Self-administrated Four private and four public schools	Questionnaire Oral clinical examination	Inclusion: Present on the day of examination. Exclusion: Not willing to participate, absent, suffering from any systemic disease that contradicts oral examination.	Impact prevalence: 36.5% Mean C-OIDP score for eating: 2.5	Medium/17
**Moreno Ruiz et al.,** **2014** **Chile** [33]	To evaluate the oral health-related quality of life using the Child-OIDP index in schoolchildren from 11–14 years old in Licantén, 2013.	Sample selection method not stated *n* = 203	11–14 years old Male: 48.3 Female: 52.7	C-OIDP Self-administrated The only school and high school	Questionnaire	Inclusion: Between first grade and fifth grade.	Impact prevalence: 68% Mean C-OIDP score: 6.92	Medium/15
**Paredes- Martínez** **et al.,** **2014** **Peru** [34]	To determine how oral conditions impact the quality of life related to oral health (HRQL) in a group of 11 and 12-year-old schoolchildren from the district of San Juan de Miraflores, Lima, in 2013.	Sample selection method not stated *n* = 169	11–12 years old Male: 49.7 Female: 50.3	C-OIDP Self-completion: List of pathologies Interview administered Educational institution	Questionnaire	Inclusion: 11 and 12-year-old schoolchildren, apparently healthy, both sexes, with authorization from the educational institution, parents’ and children’s informed consent. Exclusion: Uncorrected visual and hearing disabilities.	Impact prevalence: 100% Mean C-OIDP score: Not stated	Medium/16
**Pavithran** **et al.,** **2020** **India** [35]	To assess and compare the oral health status and impact of oral diseases on daily activities among 12 to 15-year-old institutionalized orphans and non-orphan children in Bengaluru.	Simple random sampling technique for orphanage participants. Convenience selection for non-orphanage participants. *n* = 420	12–15 years old Male orphans: 51 Female orphans: 49 Male non–orphans: 50.5 Female non–orphans: 49.5	C-OIDP Guided interviews 15 orphanages and 15 government schools	Questionnaire Oral clinical examination	Inclusion: Orphans aged 12–15 years old, consent by institutional authorities; non–orphans aged 12–15 years old with parent/guardian’s informed consent. Exclusion: Any long–standing systemic disease, physical disability, or mixed dentition.	Impact prevalence: 76.3% orphans, 65.7% non-orphans Mean C-OIDP score: 3.9 orphans, 2.8 non-orphans	High/18
**Vélez-** **Vásquez** **et al.,** **2019** **Ecuador** [36]	To associate the level of dental caries experience with the level of impact of oral conditions on the quality of life related to oral health.	Random sample *n* = 118	11–12 years old Male: 47.45 Female: 52.54	C-OIDP Interview Educational institutions	Questionnaire Oral clinical examination	Inclusion: 11- and 12-year-old schoolchildren from the educational centers of the parish of Machángara from Cuenca, Ecuador in 2017.	Impact prevalence: 88.1% Mean C-OIDP score: not stated	High/19
**Alves et al.,** **2015** **Brazil** [37]	To use normative methods to compare dental caries need with the socio-dental approach in 12-year-old adolescents according to family’s living conditions in a deprived community in Brazil.	Random sampling technique *n* = 159	12 years old Male: 49.1 Female: 50.9	C-OIDP CS-C-OIDP Self-administration Face-to-face Primary healthcare (PHC)	Questionnaires Oral clinical examination	Inclusion: Living in the areas covered by the primary healthcare system of the Manguinhos community for at least six months. Exclusion: Unable to answer the questionnaire.	Impact prevalence (Generic C-OIDP): 76.1% Impact prevalence (CS-Child–OIDP): 64.8% Mean C-OIDP score: 9.66 (generic) Mean C-OIDP score: 10.95 (specific)	Medium/17
**Bernabé** **et al.,** **2007** **Peru** [38]	To determine the prevalence, intensity and extent of the impacts of oral problems in a sample of Peruvian 11–12-year-old schoolchildren, and to compare the intensity and extent of the impacts by the type of self-perceived oral problem.	Random selection *n* = 805	11–12 years old Male: 48.8 Female: 51.2	C-OIDP Individual face-to-face interview First question self-administrated (refers to list of pathologies) Four public schools linked to a health center	Questionnaire	Inclusion: 11–12-year-olds; parental consent letter; child’s written consent.	Impact prevalence: 82.0% Mean C-OIDP score: 7.8	Medium/15
**Del** **Castillo-** **López et al.,** **2014** **Peru** [39]	To determine the impact of oral conditions on HRQL, through the Child-OIDP index, in 11- and 12-year-old schoolchildren from the Canchaque and San Miguel de El Faique districts of the Huancabamba province, from the rural area of Piura, in 2010.	Sample selection method not stated *n* = 150	11–12 years old Male: 89 Female: 61	C-OIDP Self-administrated part Face-to-face interview part Six public educational Institutions (EIs)	Questionnaire	Inclusion: 11–12 years old, healthy students, both sexes, parents’ and children’s signed informed consent.	Impact prevalence: 88.7% Mean C-OIDP score: 7.05	Medium/17
**Marcelo-** **Inguza et al.,** **2015** **Peru** [40]	To measure the impact of oral conditions on the Quality of Life Related to Health (OHRQoL) in schoolchildren aged 11–12 years in the urban-marginal area of Pachacutec-Ventanilla, Callao, Lima in 2013.	Sample selection method not stated *n* = 132	11–12 years old Male: 44 Female: 56	C-OIDP Self-administrated part (refers to list of pathologies) Face-to-face interview part Primary or secondary level of an educational institution	Questionnaire	Inclusion: 11 and 12 years old, both sexes, parents’ and children’s informed consent, apparently healthy and without any chronic systemic alteration.	Impact prevalence: 100% Mean C-OIDP score: 9.71	High/17
**Naidoo** **et al.,** **2013** **South Africa** [41]	To assess the prevalence, extent and intensity of oral impacts and their relation to perceived clinical conditions in a sample of primary school children in South Africa.	Random sampling method *n* = 1665	11–13 years old Male: 47 Female: 54	C-OIDP Face-to-face interview 26 primary schools	Questionnaire Oral clinical examination	Inclusion: 11–13 years old, 26 primary schools from amongst all those in the Ugu district, Kwazulu Natal (KZN), South Africa.	Impact prevalence: 36.2% Mean C-OIDP score: Not stated	Medium/18
**Nordin et al.,** **2019** **Malaysia** [42]	To assess the oral health status, oral health behaviors and OHRQoL among 11–12-year-old OA children in the Cameron Highlands (CH), Malaysia, and to identify factors associated with their OHRQoL.	Sample selection method not stated *n* = 227	11–12 years old Male: 51.5 Female 48.5	C-OIDP Self-administrated Primary schoolchildren	Questionnaire Oral clinical examination	Exclusion: Absent and without informed consent.	Impact prevalence: 58.6% Mean C-OIDP score: 5.45	High/17
**Reinoso- Vintimilla** **et al.** **2017** **Ecuador** [43]	Evaluate the impact of oral conditions in quality of life in children between 11 to 12 years old of schools at Sayausí, Cuenca, Ecuador.	Sample selection method *not stated* *n* = 359	11–12 years-old Male: 52.37 Female: 47.63	C-OIDP Interview administrated List of pathologies: self-administrated Church’s school	Questionnaire	Inclusion: 11 and 12 years old, in apparent good general health, both sexes, with informed assent, parents informed consent. Exclusion: who did not wish to collaborate and with physical disabilities	Impact prevalence: 98,8% Mean C-OIDP score: not stated	Medium/15
**Simangwa et al.** **2020** **Tanzania** [44]	To estimate the prevalence of oral impacts and to identify important clinical- and socio-demographic covariates. In addition, this study compares Maasai and non-Maasai adolescents regarding any association of socio- demographic and clinical covariates with oral impacts on daily performances.	One-stage cluster sample design *n* = 906	12–17 years-old Male: 43.9 Female: 56.1	C-OIDP Face- to- face interviews 23 Rural public Primary schools	Questionnaire Oral clinical examination	Inclusion: 12 to 14 years old attending rural public primary schools of Monduli and Longido districts. Exclusion: attending urban and private primary schools, absents, difficulties in learning.	Impact prevalence: 15.8% Mean C-OIDP score: not stated	High/18
**Amalia** **et al.,** **2017** **Indonesia** [45]	To examine the association between SBDP performance and OHRQoL in primary schoolchildren, while also considering the impact of untreated caries and sociodemographic factors.	Convenience sample *n* = 1906	12 years old Male: 54 Female: 46	CS-C-OIDP Interview Primary public and private schools	Questionnaire Oral clinical examination	Inclusion: All 12-year-olds from both primary public and private schools. Exclusion: No written informed consent; absent children.	Eating impact prevalence: 42.4% −38.6% Impact prevalence related to caries: 56% Impact prevalence (global): Not stated Mean C-OIDP score: 1.6–6.8	Medium/16
**Athira et al.,** **2015** **India** [46]	To determine the association, if any, between OHRQoL measured using the C-OIDP index and clinical oral health measures among 12–17-year-old children of South Bangalore.	Random sampling technique *n* = 504	12–17 years old Male: 48 Female: 52	C-OIDP Self-administration Five schools	Questionnaire Oral clinical examination	Inclusion: 12–17 years old, males and females, who can read and are ready to answer the questions, fulfill the research criteria, and consent to participate in the study. Exclusion: Did not cooperate with clinical exam; systemic disease.	Eating C-OIDP: 6.9 Impact prevalence: 43.1% Mean C-OIDP score: Not stated	Low/16
**Bianco et al.,** **2009** **Italy** [47]	To use an oral health-related quality of life (OHRQoL) measure, the Child-Oral Impact on Daily Performance (Child-OIDP), to assess the prevalence, characteristics and severity of oral impacts on health and daily activities in secondary schoolchildren, and to identify determinants such as children’s sociodemographic profile, oral hygiene habits, nutrition practices and oral health conditions, such as dental caries, periodontal diseases and orthodontics, that can predict oral impacts.	Random selection *n* = 530	11–16 years old Male: 47.4 Female: 52.6	C-OIDP Interview Secondary schools	Questionnaire Oral clinical examination	Inclusion: 11–16-year-olds; parental consent form.	Impact prevalence: 66.8% Mean C-OIDP score: 1.9	High/18
**Yetkiner** **et al.,** **2014** **Turkey** [48]	(1) To determine orthodontic treatment need, self-esteem and OHRQoL of primary schoolchildren, and (2) To investigate possible influences of orthodontic treatment need on OHRQoL and self-esteem.	Sample selection method not stated *n* = 219	13–14 years old Male: 51.60 Female: 48.40	C-OIDP Self-administrated The sixth year of primary public school	Questionnaire Oral clinical examination	Inclusion: 13–14 years, no history of previous orthodontic treatment, with informed consent.	Impact prevalence: 69.9% Mean C-OIDP score for eating: 3	Medium/18

Notes: C-OIDP = Child-Oral Impact on Daily Performance, CS-C-OIDP = Condition Specific Child-Oral Impact on Daily Performance, OHRQoL = oral health-related quality of life, STROBE checklist for cross-sectional studies (STrengthening the Reporting of OBservational studies in Epidemiology), FLC 3.0: Ficha Lectura Crítica 3.0.

## 3. Results

### 3.1. Study Selection and Flow Diagram

**Figure 1:**
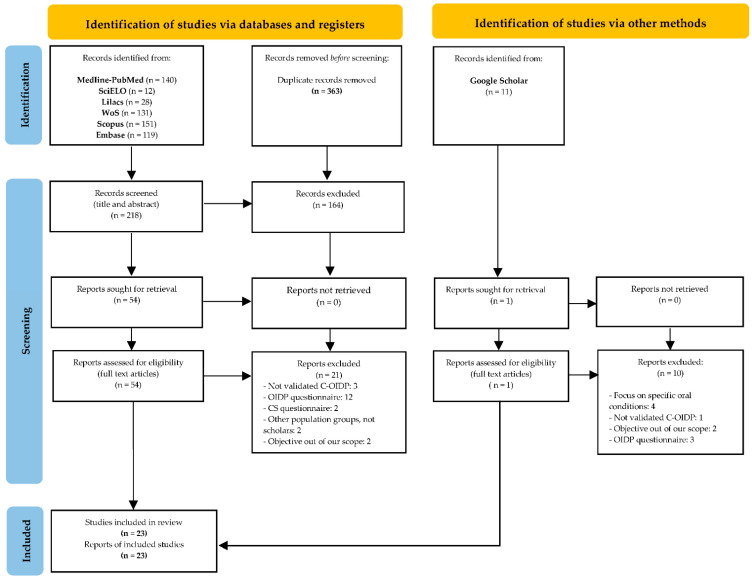
PRISMA 2020 flow diagram for new systematic reviews that included searches of databases, registers and other sources. From: Page MJ, McKenzie JE, Bossuyt PM, Boutron I, Hoffmann TC, Mulrow CD, et al. The PRISMA 2020 statement: an updated guideline for reporting systematic reviews. BMJ 2021;372:n71. doi:10.1136/bmj.n71. For more information, visit: http://www.prisma-statement.org/ (accessed on 9 September 2021).

Figure 1 (PRISMA Flow diagram) illustrates the study selection process. The electronic search identified a total of 581 articles (140 in PubMed-Medline, 12 in SciELO, 28 in Lilacs, 131 in Web of Science (WoS), 151 in Scopus and 119 in Embase). Through a manual search performed in Google Scholar, 11 additional studies were identified. After discarding duplicates, 218 articles remained. After reading the titles and abstracts, 164 articles were removed, leaving a total of 54 full-text articles assessed for eligibility.

After detailed analysis of these 54 articles, another 31 articles were excluded for the following reasons: use of a non-validated C-OIDP (4); use of the OIDP questionnaire (15); use of a condition-specific questionnaire (2); focused on specific oral condition (4), other population groups, not scholars (2); objective out of our scope (4) (Figure S4). After the study selection process, the final number of studies included for the qualitative synthesis was 23.

### 3.2. Qualitative Synthesis

#### 3.2.1. Population (P)

Among the 23 articles screened, the total number of study participants was 12,604, aged between 11 and 17 years old. Most studies included both sexes, although one study included only male subjects [26]. In total, there were approximately 52% female participants and 48% males. Among the 23 articles, distinct population groups were identified as urban in ten studies [27,28,29,30,31,32,33,34,35,36], rural in eight studies [37,38,39,40,41,42,43,44] and mixed or not specified in five studies [26,45,46,47,48]. The sample size varied between 118 [36] and 1906 [45] participants.

The sample selection method was a random sampling technique in most studies, convenience sample in one study [45] and another study used a mixed technique (random sampling in one part of the sample and convenience sample in another part) [35]. The sampling method was not stated in seven studies [33,34,39,40,42,43,48].

#### 3.2.2. Intervention (I) (Child-OIDP Questionnaire)

The type of questionnaire used was the generic C-OIDP one in most articles; one study used only the CS-C-OIDP (condition-specific) [45] and another study used both the generic and condition-specific inventories [37]. The type of intervention included administration of the OHRQoL questionnaire in seven articles [30,33,34,38,39,40,43] and both the OHRQoL questionnaire and oral clinical examination in all other studies [26,27,28,29,31,32,35,36,37,41,42,44,45,46,47,48].

The mode of administration of the questionnaire was self-administrated in five articles [32,33,42,46,48], interview-administered in 12 publications [26,28,30,31,34,35,36,41,43,44,45,47] and a mixed system (self-administrated questions and interview-administered questions) in six articles [27,29,37,38,39,40].

Questionnaires were administered in schools in all the studies except one, where participants from orphanages were included [35]. Inclusion and exclusion criteria were clearly stated in the majority of studies, though not completely specified in three studies [33,36,41].

#### 3.2.3. Outcome (O)

Results were specified as the overall impact prevalence in most studies, and one study reported the impact prevalence for each of the specific dimensions [45]. The mean C-OIDP score was reported in the majority of publications, although it was not present in seven articles [31,34,36,41,43,44,46], while two authors provided mean C-OIDP scores that referred to specific affected dimensions [32,48].

The most frequently affected dimensions were eating [26,28,29,30,31,32,33,34,35,36,37,38,39,40,41,42,44,45,47,48], teeth brushing [27,46] and the emotional state [43].

The intensity of the impact was found to be low in most publications. Two authors reported a high intensity in 25% of the sample [37,38], mainly affecting psychosocial dimensions such as the emotional state and going to school [41], while Nordin et al. [42] found that 4.6% of the sample reported a high intensity of impact on smiling.

The extent of the impact was below 3.9 affected dimensions in the last three months in a rural setting [38,39,40,41], while the mean PWI (performances with impact) in an urban setting was up to 4.8 [28,34,36,43], with the exception of Dumitrache et al. [31] who reported up to seven affected dimensions.

### 3.3. Risk of Bias within Studies and Quality Assessment

According to the individual evidence table filled in for each study, and STROBE checklist compliance, the risk of bias was found to be low in eight studies and medium in 15 studies.

With regard to the quality assessment, it was found to be low in two studies, medium in 13 studies and high in another eight studies.

## 4. Discussion

The aim of this systematic review was to identify cross-sectional descriptive studies on OHRQoL in adolescents using the C-OIDP questionnaire in a school population aged between 11 and 18 years, carried out in the last 17 years, and to analyze their methodologies and the most relevant results reported. This time range was chosen because the C-OIDP measurement instrument was developed in 2004 [17], with subsequent translation and validation in other languages and cultural contexts starting in 2005.

Regarding the methodology followed in the studies included in the review, the most frequent objective was to determine the relationship between OHRQoL and oral health status, and secondly to assess the impact of oral conditions on OHRQoL. Bianco [47] and Simangwa [44] evaluated the importance of sociodemographic factors on oral impact in adolescents, while Amalia et al. [45] also analyzed the relationship between the child oral health program implemented at a scholar level and OHRQoL.

Alves [37] compared normative needs with perceived needs in oral health service planning, and Athira [46] looked at the relationship between an SBDP (school-based dental program) and OHRQoL. Yetkiner et al. [48] studied the relationship between orthodontic treatment needs and OHRQoL.

The reviewed articles had a cross-sectional design, and although the implementation of the STROBE methodology was reported in only one of them [35], 83% of the studies included more than 16 of the total 22 items that make up the STROBE checklist. The methodological quality assessed, according to the evidence table based on FLC 3.0, was low in two studies, medium in 13 publications and high in eight articles.

Interestingly, the time period was not described in seven studies [31,36,37,41,45,46,48], which is an important element to include in descriptive studies.

Although the WHO recommends 12 years as the indexed age for studies in this population group, most studies also included participants older than 12 years of age [49].

The sociodemographic characteristics of the sample included a rural population in eight studies [37,38,39,40,41,42,43,44], urban in ten studies [27,28,29,30,31,32,33,34,35,36] and mixed or unspecified in five studies [26,45,46,47,48]. From the reviewed studies, it can be deduced that to characterize the population sample, it is necessary to specify the socioeconomic level, describe whether the population is urban, rural or semi-urban and outline the level of access to oral health services, to interpret the results obtained, since these characteristics influence the health status and perception of health [40].

The sample selection method was reported as simple random sampling in 14 studies [26,27,28,29,30,31,32,35,36,37,38,41,46,47]. Although the absence of random sampling techniques makes it difficult to apply the results to the general population and decreases the external validity, only some authors included a statement in their discussion on the impossibility of applying their results to the general population [26,37,38,40,41,42].

The sample size ranged from 118 [36] participants to 1906 [45], with a total of 12,604 participants. Only six authors [28,32,38,42,44,46] justified the size based on the expected impact prevalence, precision and assumed type-1 error.

Statistical analysis was fully described in most articles, with the Chi square, Kruskal–Wallis and Mann–Whitney U-test being the most frequently used in the analysis of the data.

In relation to the most relevant results reported, the prevalence of impact was high in all the analyzed studies, including rural and urban populations, as well as in the studies where the sample setting was not specified. A high prevalence of impact associated with a low socioeconomic status was reported, except for the studies carried out by Naidoo [41] and Simangwa [44] in low socioeconomic populations. The first author found a prevalence of 36% in South Africa related to DMFT = 0, while the second author found an impact prevalence of 15% in Tanzania related to DMFT = 1 and the traditional way of life of the Maasai population where there is also a high prevalence of dental fluorosis.

Athira [46] in India reported a moderate impact prevalence of 43% on the eight dimensions assessed by the questionnaire, which the author interpreted as being due to memory bias in adolescents since several impact-causing oral conditions are of short duration and are quickly forgotten by the subject.

Alzahrani et al. [26], in a sample of only males, obtained an impact prevalence of 75%, which contrasts with other authors who found a higher prevalence of impact among females.

In urban populations, Basavaraj (India) [28], Pavithran (India) [35] and Do (Vietnam) [30] found an association between age and the C-OIDP index: the younger the age, the greater the impact on oral quality of life. They agree with Gherunpong [17], who found that younger children are more sensitive to oral symptoms than teenagers.

In studies with participants aged 12 years or younger [29,34,36,37,38,39,40,42,43,45], an impact prevalence between 100% and 58.6% was observed, and a mean C-OIDP score between 9.7 and 5.4. In contrast, in studies with participants over 12 years of age (the other 13 studies), the impact prevalence was between 87% and 15.8%, lower than in younger adolescents. And in the latter age group, the mean C-OIDP score was lower than 2.8 in half of the studies reporting it. Altogether, the results suggest that the impact on oral quality of life is greater in younger adolescents.

In the studies carried out in rural areas, no significant differences were found by sex in terms of the prevalence, severity, intensity and extent of oral impacts, while in urban areas, only Pavithran [35], Castro [29] and Moreno [33] found differences by sex, reporting that the mean C-OIDP score is higher in females than in males.

Amalia [45] and Bianco [47], in populations with unspecified demographic settings, found an association between being female and reporting a worse oral quality of life in adolescents. In total, this association appears in five of the 23 analyzed studies in this systematic review.

The most frequently affected dimension in all the studies in both rural and urban settings was eating, concurring with previous studies. The least affected dimension was socializing [38,39,40,44]. Toothache was perceived as the first cause of impact in several studies [38,39,40,45] and bleeding gums only in Nordin [42] in an indigenous Malaysian minority with a high level of caries and periodontal disease.

Bakhtiar [27] and Athira [46] found that the most frequently affected dimension was toothbrushing, and in Reinoso’s study [43], it was the emotional state.

Among the oral conditions reported by the participants as having an impact on their oral quality of life, analyzed by dimensions: the impacts on toothbrushing were primarily due to bleeding gums, the impacts on smiling were due to tooth color and position, the impacts on eating were due to caries and bleeding gums, while halitosis was reported as the first cause of impact in Bakhtiar (Iran) [27] and tooth color and position in Moreno (Chile) [33].

In the analysis of the association between clinical status and the C-OIDP index, a significant association was seen between previous caries experience, DMFT, caries in primary teeth, aphthous ulcers, fluorosis (Athira [46] found that the higher the degree of fluorosis, the greater the impact, while Simangwa [44] observed the opposite), bleeding gums, malocclusion and the value of the C-OIDP index. Meanwhile, Vélez [36] found that there is an inverse relationship between the DMFT variables and the C-OIDP index (the greater the DMFT, the lower the C-OIDP). This concurs with the paradox of dental need defined by Adunola [50]: those who do not receive dental care are those who have the greatest need. From DMFT = 7 onwards, the impact on oral quality of life decreases or is null.

As for the influence of behavioral factors on quality of life, an association was found between better oral hygiene and a lower C-OIDP index. In addition, Bianco [47] reported an association between low fruit intake and frequent use of mouthwash and an increase in the C-OIDP index.

Among the limitations of this systematic review, we are aware of a possibility of publication bias due to the eligibility criteria followed, meaning otherwise useful studies might have been excluded. Also, the inclusion criteria may have led to unintended selection bias. We have tried to detect publication bias through the comprehensive literature search strategy. The use of the FLC 3.0-based evidence table with a qualitative scale to assess the quality of studies may also entail the risk of bias on the part of the evaluator. Selected studies demonstrated substantial variability; the main sources of heterogeneity included the mean C-OIDP score being missing in many articles, the C-OIDP score often reported related to specific dimensions and not as an overall score, differing outcome measures, major variations in the sample selection method, and assessment of the intensity and severity of the impact on OHRQoL inconsistently reported in some studies. The heterogeneity of results must be taken into account when assessing the data.

## 5. Conclusions

In conclusion, in the cross-sectional studies of OHRQoL in the adolescent school populations included in this review, using the validated C-OIDP questionnaire, the OHRQoL perceived by adolescents is related to age, sex and sociodemographic factors. Eating was the most frequently affected dimension and toothache was the first cause of impact, showing a generally mild intensity and severity of impact, with the average number of affected dimensions between 1 and 4.8. Previous caries experience, the DMFT index, caries in primary teeth, canker sores, bleeding gums and malocclusion have been reported as factors associated with a lower level of OHRQoL. Longitudinal studies are needed in the future to complete the information obtained in the cross-sectional studies conducted to date.

## Figures and Tables

**Table 1:** Inclusion and exclusion criteria for the systematic review.

Criteria	Inclusion	Exclusion
1. Study design	Cross-sectional studies	Systematic reviews, metanalysis, literature reviews, case-control studies, case-series, cohort’s studies, reports, papers, conference proceedings
2. Population	Adolescents	Adults
3. Population age range	11–18 years-old	<11-years-old >18-years-old
4. Administered questionnaire	C-OIDP/OIDP	Other oral health-related quality of life-validated questionnaires
5. Year of publication	Last 17 years (2005–2021)	<2005
6. Language	English, Spanish	Other languages
7. Publication type	Original articles, full-text	Not original articles, abstracts

Note: C-OIDP = Child Oral Impact on Daily Performance.

## Data Availability

Not applicable.

## Supplementary Materials

The following are available online at https://www.mdpi.com/article/10.3390/ijerph182412995/s1, Figure S1: PRISMA Guidelines 2020, Figure S2: PROSPERO registration, Figure S3: Search strategy 28/7/21, Figure S4: Articles excluded and reasons, Table S1: STROBE 23 articles, Table S2: Evidence tables.

## Abbreviations

OHRQoL = oral health-related quality of life; C-OIDP = Child Oral Impact on Daily Perfor-mance; CS-C-OIDP = Condition Specific Child-Oral Impact on Daily Performance; WHO = World Health Organization; STROBE checklist for cross-sectional studies (STrengthening the Reporting of OBservational studies in Epidemiology); FLC 3.0: Ficha Lectura Crítica 3.0.
