# Supplementary material for: Oral Health-Related Quality of Life in Adolescents as Measured with the Child-OIDP Questionnaire: A Systematic Review
Source: Int J Environ Res Public Health. 2021 Dec 9;18(24):12995. doi: 10.3390/ijerph182412995 (PMC8701449; doi:10.3390/ijerph182412995)
Supplement: Supplementary file 1 [file ijerph-18-12995-s001.zip › ijerph-1477806-supplementary.pdf]

International Journal of
Environmental Research and Public Health

Supplementary Material

Figure S1: PRISMA Guidelines 2020.

Figure S2: Prospero Registration.

Figure S3: Search strategy 28/7/21.

Figure S4: Articles excluded and reasons for exclusion.

Table S1: Strobe 23 articles.

Table S2: Evidence tables.

Section and Topic	Item #	Checklist item	Location where item is reported
**TITLE**			
Title	1	Identify the report as a systematic review.	p. 1
**ABSTRACT**			
Abstract	2	See the PRISMA 2020 for Abstracts checklist.	p. 1
**INTRODUCTION**			
Rationale	3	Describe the rationale for the review in the context of existing knowledge.	pp. 1-2
Objectives	4	Provide an explicit statement of the objective(s) or question(s) the review addresses.	p. 2
**METHODS**			
Eligibility criteria	5	Specify the inclusion and exclusion criteria for the review and how studies were grouped for the syntheses.	p. 3
Information sources	6	Specify all databases, registers, websites, organisations, reference lists and other sources searched or consulted to identify studies. Specify the date when each source was last searched or consulted.	pp. 2-3
Search strategy	7	Present the full search strategies for all databases, registers and websites, including any filters and limits used.	pp. 2-3
Selection process	8	Specify the methods used to decide whether a study met the inclusion criteria of the review, including how many reviewers screened each record and each report retrieved, whether they worked independently, and if applicable, details of automation tools used in the process.	pp. 2-3

Section and Topic	Item #	Checklist item	Location where item is reported
Data collection process	9	Specify the methods used to collect data from reports, including how many reviewers collected data from each report, whether they worked independently, any processes for obtaining or confirming data from study investigators, and if applicable, details of automation tools used in the process.	pp. 3-4
Data items	10a	List and define all outcomes for which data were sought. Specify whether all results that were compatible with each outcome domain in each study were sought (e.g. for all measures, time points, analyses), and if not, the methods used to decide which results to collect.	p. 4
	10b	List and define all other variables for which data were sought (e.g. participant and intervention characteristics, funding sources). Describe any assumptions made about any missing or unclear information.	p. 4<br>pp. 7-16
Study risk of bias assessment	11	Specify the methods used to assess risk of bias in the included studies, including details of the tool(s) used, how many reviewers assessed each study and whether they worked independently, and if applicable, details of automation tools used in the process.	p. 4
Effect measures	12	Specify for each outcome the effect measure(s) (e.g. risk ratio, mean difference) used in the synthesis or presentation of results.	
Synthesis methods	13a	Describe the processes used to decide which studies were eligible for each synthesis (e.g. tabulating the study intervention characteristics and comparing against the planned groups for each synthesis (item #5)).	
	13b	Describe any methods required to prepare the data for presentation or synthesis, such as handling of missing summary statistics, or data conversions.	
	13c	Describe any methods used to tabulate or visually display results of individual studies and syntheses.	p. 4
	13d	Describe any methods used to synthesize results and provide a rationale for the choice(s). If meta-analysis was performed, describe the model(s), method(s) to identify the presence and extent of statistical heterogeneity, and software package(s) used.	
	13e	Describe any methods used to explore possible causes of heterogeneity among study results (e.g. subgroup analysis, meta-regression).	
	13f	Describe any sensitivity analyses conducted to assess robustness of the synthesized results.	
Reporting bias assessment	14	Describe any methods used to assess risk of bias due to missing results in a synthesis (arising from reporting biases).	
Certainty	15	Describe any methods used to assess certainty (or confidence) in the body of evidence	

Section and Topic	Item #	Checklist item	Location where item is reported
assessment		for an outcome.	
**RESULTS**			
Study selection	16a	Describe the results of the search and selection process, from the number of records identified in the search to the number of studies included in the review, ideally using a flow diagram.	pp. 4-6
	16b	Cite studies that might appear to meet the inclusion criteria, but which were excluded, and explain why they were excluded.	pp. 4-5
Study characteristics	17	Cite each included study and present its characteristics.	pp. 7-17
Risk of bias in studies	18	Present assessments of risk of bias for each included study.	pp. 17
Results of individual studies	19	For all outcomes, present, for each study: (a) summary statistics for each group (where appropriate) and (b) an effect estimate and its precision (e.g. confidence/credible interval), ideally using structured tables or plots.	
Results of syntheses	20a	For each synthesis, briefly summarise the characteristics and risk of bias among contributing studies.	
	20b	Present results of all statistical syntheses conducted. If meta-analysis was done, present for each the summary estimate and its precision (e.g. confidence/credible interval) and measures of statistical heterogeneity. If comparing groups, describe the direction of the effect.	
	20c	Present results of all investigations of possible causes of heterogeneity among study results.	
	20d	Present results of all sensitivity analyses conducted to assess the robustness of the synthesized results.	
Reporting biases	21	Present assessments of risk of bias due to missing results (arising from reporting biases) for each synthesis assessed.	
Certainty of evidence	22	Present assessments of certainty (or confidence) in the body of evidence for each outcome assessed.	
**DISCUSSION**			
Discussion	23a	Provide a general interpretation of the results in the context of other evidence.	p. 18-19
	23b	Discuss any limitations of the evidence included in the review.	p. 20
	23c	Discuss any limitations of the review processes used.	p. 20
	23d	Discuss implications of the results for practice, policy, and future research.	p. 19

Section and Topic	Item #	Checklist item	Location where item is reported
**OTHER INFORMATION**			
Registration and protocol	24a	Provide registration information for the review, including register name and registration number, or state that the review was not registered.	p. 2
	24b	Indicate where the review protocol can be accessed, or state that a protocol was not prepared.	p. 2
	24c	Describe and explain any amendments to information provided at registration or in the protocol.	
Support	25	Describe sources of financial or non-financial support for the review, and the role of the funders or sponsors in the review.	p. 20
Competing interests	26	Declare any competing interests of review authors.	p. 20
Availability of data, code and other materials	27	Report which of the following are publicly available and where they can be found: template data collection forms; data extracted from included studies; data used for all analyses; analytic code; any other materials used in the review.	

Figure S1. PRISMA Guidelines 2020.

CRD-REGISTER <irss505@york.ac.uk> 24/12/2020 07:01

PROSPERO Registration message [222392]

A rossanagreco93@libero.it

Dear Dr Greco,

We apologise for the delay in dealing with your registration, an ever-increasing number of applications has led to a backlog and substantial delays for some users.

PROSPERO is currently prioritising submissions related to COVID-19. To enable us to focus on these submissions, and to avoid additional delay, during the pandemic we will automatically publish submissions that have been waiting more than 30 days for registration.

This applies to your systematic review "Oral health related quality of life in adolescents. A systematic review" which was published on our website on Dec 24, 2020.

The records will be published exactly as submitted, without review by the PROSPERO team, so the public record will indicate:

"To enable PROSPERO to focus on COVID-19 registrations during the 2020 pandemic, this registration record was automatically published exactly as submitted. The PROSPERO team has not checked eligibility"

Review owners have always been responsible for the quality and content of PROSPERO records, and high-quality well-written records will continue to speak for themselves.

Your registration number is: CRD42020222392

You are free to update the record at any time, all submitted changes will be displayed as the latest version with previous versions available to public view. Please also give brief details of the key changes in the Revision notes facility and remember to update your record when your review is published. You can log in to PROSPERO and access your records at https://www.crd.york.ac.uk/PROSPERO

Best wishes for the successful completion of your review.

Yours sincerely,

PROSPERO Administrator
Centre for Reviews and Dissemination
University of York
York YO10 5DD
t: +44 (0) 1904 321049
e: CRD-register@york.ac.uk
www.york.ac.uk/inst/crd

PROSPERO is funded by the National Institute for Health Research and produced by CRD, which is an academic department of the University of York.

Email disclaimer: https://www.york.ac.uk/docs/disclaimer/email.htm

Other non-commercial resources that may be of interest
SRDR-Plus is a systematic review data management and archival tool that is available free of charge
http://srdrplus.ahrq.gov.

Figure S2. Prospero Registration.

	Database	Search strategy	Findings
28/07/2021	**Medline (PubMed)**	#**1** oral health quality of life ("oral health"[MeSH Terms] OR ("oral"[All Fields] AND "health"[All Fields]) OR "oral health"[All Fields]) AND ("quality of life"[MeSH Terms] OR ("quality"[All Fields] AND "life"[All Fields]) OR "quality of life"[All Fields])	**12,951**
		#**2** adolescents or children or scholars "adolescences"[All Fields] OR "adolescency"[All Fields] OR "adolescent"[MeSH Terms] OR "adolescent"[All Fields] OR "adolescence"[All Fields] OR "adolescents"[All Fields] OR "adolescent s"[All Fields] OR "child"[MeSH Terms] OR "child"[All Fields] OR "children"[All Fields] OR "child s"[All Fields] OR "children s"[All Fields] OR "childrens"[All Fields] OR "childs"[All Fields] OR "scholar"[All Fields] OR "scholars"[All Fields] OR "scholars"[All Fields]	**3,994,971**
		#**3** Child-OIDP or OIDP "Child-OIDP"[All Fields] OR "OIDP"[All Fields]	**298**
		#**1** AND #**2** AND #**3** ("oral health"[MeSH Terms] OR ("oral"[All Fields] AND "health"[All Fields])OR "oral health"[All Fields]) AND ("quality of life"[MeSH Terms] OR("quality"[All Fields] AND "life"[All Fields]) OR "quality of life"[All Fields])AND ("adolescences"[All Fields] OR "adolescency"[All Fields] OR"adolescent"[MeSH Terms] OR "adolescent"[All Fields] OR	

		"adolescence"[All Fields] OR "adolescents"[All Fields] OR "adolescent s"[All Fields] OR("child"[MeSH Terms] OR "child"[All Fields] OR "children"[All Fields] OR"child s"[All Fields] OR "children s"[All Fields] OR "childrens"[All Fields] OR"childs"[All Fields]) OR ("scholar"[All Fields] OR "scholar s"[All Fields] OR"scholars"[All Fields])) AND ("Child-OIDP"[All Fields] OR "OIDP"[All Fields])	**<u>140</u>**
28/07/2021	**Scopus**	#**1** TITLE-ABS-KEY (oral AND health AND quality AND of AND life)	**14,292**
		#**2** TITLE-ABS-KEY (adolescents OR children OR scholars)	**4,641,473**
		#**3** TITLE-ABS-KEY (Child-OIDP OR OIDP)	**329**
		#**1** TITLE-ABS-KEY (oral AND health AND quality AND of AND life) AND #**2** TITLE-ABS-KEY (adolescents OR children OR scholars) AND #**3** TITLE-ABS-KEY (Child-OIDP OR OIDP)	**<u>151</u>**
28/07/2021	**Wos (Web of Science)**	#**1** TS= oral health quality of life Indexes=SCI-EXPANDED, SSCI, A&HCI, CPCI-S, CPCI-SSH, BKCI-S, BKCI-SSH, ESCI, CCR-EXPANDED, IC Timespan=All years	**11,204**
		#**2** TS= (adolescents or children or scholars) Indexes=SCI-EXPANDED, SSCI, A&HCI, CPCI-S, CPCI-SSH, BKCI-S, BKCI-SSH, ESCI, CCR-EXPANDED, IC Timespan=All years	**2,255,061**
		#**3** TS= (Child-OIDP or OIDP)	

		Indexes=SCI-EXPANDED, SSCI, A&HCI, CPCI-S, CPCI-SSH, BKCI-S, BKCI-SSH, ESCI, CCR-EXPANDED, IC Timespan=All years	**288**
		#**1** AND #**2** AND #**3** Indexes=SCI-EXPANDED, SSCI, A&HCI, CPCI-S, CPCI-SSH, BKCI-S, BKCI-SSH, ESCI, CCR-EXPANDED, IC Timespan=All years	**131**
28/07/2021	**Embase**	#**1** oral health quality of life<br>oral AND health AND quality AND of AND life	**30,016**
		#**2** adolescents or children or scholars<br>adolescents OR children OR scholars	**3,892,153**
		#**3** Child-OIDP or OIDP<br>Child-OIDP OR OIDP	**283**
		#**1** AND #**2** AND #**3**<br>oral AND health AND quality AND of AND life AND (adolescents OR children OR scholars) AND (Child-OIDP OR OIDP)	**119**
28/07/2021	**Lilacs**	#**1** oral health quality of life<br>oral health quality of life	**1,494**
		#**2** adolescents or children or scholars<br>adolescents OR children OR scholars	**154,464**
		#**3** Child-OIDP or OIDP<br>Child-OIDP OR OIDP	**73**
		#**1** AND #**2** AND #**3**	

		(oral health quality of life) AND (adolescents or children or scholars) AND (Child-OIDP or OIDP)	**28**
28/02/2021	**SciELO**	#**1** oral health quality of life<br>oral health quality of life	**651**
		#**2** adolescents or children or scholars<br>adolescents or children or scholars	**50,739**
		#**3** Child-OIDP or OIDP<br>Child-OIDP or OIDP	**43**
		#**1** AND #**2** AND #**3**<br>(oral health quality of life) AND (adolescents OR children OR scholars) AND (Child-OIDP OR OIDP)	**12**
			**TOTAL 581**

Figure S3. Search Strategy 28/7/21.

	Author / Year	Article Title	Reason for exclusion
1	*Bernabe (2009)*	Impacts on daily performances attributed to malocclusions by British adolescents	OIDP questionnaire
2	*Chukwumah (2016)*	Impact of dental caries and its treatment on the quality of life of 12- to 15-year-old adolescents in Benin, Nigeria	Focus on specific oral conditions (Dental caries focused)
3	*Freitas (2014)*	Association between dental caries activity, quality of life and obesity in Brazilian adolescents	Focus on specific oral conditions (Dental caries focused)
4	*Herkrath (2013)*	Comparison of normative methods and the socio-dental approach to assessing orthodontic treatment needs in 12-year-old schoolchildren	Focus on specific oral conditions (Orthodontics)
5	*Krisdapong (2013)*	Sociodemographic differences in oral health-related quality of life related to dental caries in Thai school children	OIDP questionnaire
6	*Krisdapong (2013)*	Impacts on quality of life related to dental caries in a national representative sample of Thai 12-and 15-year-olds	OIDP questionnaire
7	*Krisdapong (2014)*	Associations between perceived needs for dental treatment, oral health-related quality of life and oral diseases in school-aged Thai children	OIDP questionnaire
8	*Krisdapong (2012)*	The impacts of gingivitis and calculus on Thai children's quality of life	OIDP questionnaire
9	*Krisdapong (2014)*	Which aspects of an oral health-related quality of life measure are mainly associated with global ratings of oral health in children	OIDP questionnaire
10	*Krisdapong (2012)*	Impacts of recurrent aphthous stomatitis on quality of life of 12- and 15-year-old Thai children	OIDP questionnaire
11	*Krisdapong (2012)*	Setting oral health goals that include oral health-related quality of life measures: a study carried out among adolescents in Thailand	Condition specific (CS) questionnaire
12	*Krisdapong (2012)*	Using associations between oral diseases and oral health-related quality of life in a nationally	Objective out of our scope (Oral disease and health planning)

		representative sample to propose oral health goals for 12-year-old children in Thailand	
**13**	***Krisdapong (2012)***	Relationships between oral diseases and impacts on Thai schoolchildren's quality of life: Evidence from a Thai national oral health survey of 12- and 15-year-olds	OIDP questionnaire
**14**	***Krisdapong (2009)***	Oral health-related quality of life of 12-and 15-year-old Thai children: Findings from a national survey	OIDP questionnaire
**15**	***Masjedi (2019)***	Relationship between malocclusion and oral health related quality of life among high school girl students in Ahvaz-lran	OIDP questionnaire
**16**	***Mbawalla (2011)***	Discriminative ability of the generic and condition-specific Child-Oral Impacts on Daily Performances (Child-OIDP) by the Limpopo-Arusha School Health (LASH) Project: A cross-sectional study	Condition specific (CS) questionnaire
**17**	***Mbwalla (2019)***	Behavioural and sociodemographic determinants of oral health-related quality of life among adolescents in Zanzibar, Tanzania	Not validated C-OIDP version
**18**	***Oliveira (2020)***	Oral health-related quality of life among 12-year-olds: results from SB-Minas Gerais	OIDP questionnaire
**19**	***Pasiga (2018)***	Socio-dental and family living condition approach for planning dental care: A Cross-Sectional study among Indonesian students	Objective out of our scope
**20**	***Pau (2008***	Dental pain and care-seeking in 11-14-yr-old adolescents in a low-income country	Not validated C-OIDP version (Modified C-OIDP inventory applied)
**21**	***Pavlovic (2019)***	Oral hygiene habits and prosthodontic treatment needs in younger adolescent population of pančevo, Serbia	OIDP questionnaire
**22**	***Pentapati (2013)***	Oral health impact, dental caries, and oral health behaviours among the National Cadets Corps in South India	Other population group (National cadets corps)

23	***Perera (2010)***	Social inequality in perceived oral health among Sri Lankan adolescents	OIDP questionnaire
24	***Prasertsom (2020)***	Condition-Specific Oral Health Impacts in Thai Children and Adolescents: Findings From the National Oral Health–Related Quality of Life Survey	OIDP questionnaire
25	***Ramos Jorge (2014)***	Impact of treated/untreated traumatic dental injuries on quality of life among Brazilian schoolchildren	Focus on specific oral conditions (Traumatic dental injuries)
26	***Ravaghi (2019)***	Socioeconomic Variation in the association between Malocclusion and Oral Health Related Quality of Life	Not validated C-OIDP version
27	***Silva Souza (2018)***	Impact of untreated dental caries on the daily activities of children	OIDP questionnaire
28	***Sudeep (2014)***	Oral Health Related Quality of Life among 12-15 Year Old Children Residing at Orphanages in South India- A Descriptive Study	Objective out of our scope
29	***Tagelsir (2013)***	Oral health of visually impaired schoolchildren in Khartoum State, Sudan	Other populations group (visually impaired children)
30	***Tsakos (2006)***	Can oral health-related quality of life measures substitute for normative needs assessments in 11 to 12-year-old children?	Objective out of our scope (validation study)
31	***Wu (2021)***	Associated Factors of Oral Health-related Quality of Life in Chinese Adolescents Aged 12-15 Years	Not validated C-OIDP version

Figure S4. Articles excluded after full text analysis and reasons for exclusion.

Reasons for exclusion:

- Not validated C-OIDP: **4**
- OIDP questionnaire: **15**
- CS questionnaire: **2**
- Focus on specific oral conditions (dental caries, orthodontics, traumatic dental injuries): **4**
- Other population groups, not scholars (National cadets corps, visually impaired children): **2**
- Objective out of our scope: **4**

Table S1. STROBE 23 articles.

STROBE CHECKLIST *CROSS-SECTIONAL STUDIES* - ARTICLE 1

TITLE: Planning oral health care using the sociodental approach and the index of family living conditions: a cross-sectional study in Brazilian adolescents

Alves et al. (2015)

Item		Recommendation	Page n°
1	**Title and Abstract**	**(a)** Indicate the study's design with a commonly used term in the title or the abstract	1
		**(b)** Provide in the abstract an informative and balanced summary of what was done and what was found	1
	**Introduction**		
2	*Background/Rationale*	Explain the scientific background and rationale for the investigation being reported	2
3	*Objectives*	State specific objectives, including any prespecified hypotheses	2
	**Methods**		
4	*Study design*	Present key elements of study design early in the paper	2
5	*Setting*	Describe the setting, locations, and relevant dates, including periods of recruitment, exposure, follow-up, and data collection	3
6	*Participants*	**(a)** Give the eligibility criteria, and the sources and methods of selection of participants	3
7	*Variables*	Clearly define all outcomes, exposures, predictors, potential confounders, and effect modifiers. Give diagnostic criteria, if applicable	3-4
8	*Data sources/ measurement*	For each variable of interest, give sources of data and details of methods of assessment (measurement). Describe comparability of assessment methods if there is more than one group	4
9	*Bias*	Describe any efforts to address potential sources of bias	
10	*Study size*	Explain how the study size was arrived at	4
11	*Quantitative variables*	Explain how quantitative variables were handled in the analyses. If applicable, describe which groupings were chosen and why	
12	*Statistical methods*	**(a)** Describe all statistical methods, including those used to control for confounding	3-5
		**(b)** Describe any methods used to examine subgroups and interactions	4
		**(c)** Explain how missing data were addressed	
		**(d)** If applicable, describe analytical methods taking account of sampling strategy	4
		**(e)** Describe any sensitivity analyses	
	**Results**		
13	*Participants*	**(a)** Report numbers of individuals at each stage of study—eg numbers potentially eligible, examined for eligibility, confirmed eligible, included in the study, completing follow-up, and analysed	
		**(b)** Give reasons for non-participation at each stage	
		**(c)** Consider use of a flow diagram	
14	*Descriptive data*	**(a)** Give characteristics of study participants (eg demographic, clinical, social) and information on exposures and potential confounders	5

		**(b)** Indicate number of participants with missing data for each variable of interest	
**15**	*Outcome data*	Report numbers of outcome events or summary measures	5-7
**16**	*Main results*	**(a)** Give unadjusted estimates and, if applicable, confounder-adjusted estimates and their precision (eg, 95% confidence interval). Make clear which confounders were adjusted for and why they were included	5
		**(b)** Report category boundaries when continuous variables were categorized	6
		**(c)** If relevant, consider translating estimates of relative risk into absolute risk for a meaningful time period	
**17**	*Other analyses*	Report other analyses done—eg analyses of subgroups and interactions, and sensitivity analyses	
	**Discussion**		
**18**	*Key results*	Summarise key results with reference to study objectives	7-8
**19**	*Limitations*	Discuss limitations of the study, taking into account sources of potential bias or imprecision. Discuss both direction and magnitude of any potential bias	9
**20**	*Interpretation*	Give a cautious overall interpretation of results considering objectives, limitations, multiplicity of analyses, results from similar studies, and other relevant evidence	9
**21**	*Generalisability*	Discuss the generalisability (external validity) of the study results	9
	**Other information**		
**22**	Funding	Give the source of funding and the role of the funders for the present study and, if applicable, for the original study on which the present article is based	
**TOT.**			17

STROBE CHECKLIST *CROSS-SECTIONAL STUDIES* - ARTICLE 2

TITLE: Association between oral diseases and impact on daily performance among male Saudi schoolchildren

Alzahrani et al.(2019)

**Item**		**Recommendation**	**Page n°**
**1**	**Title and Abstract**	**(a)** Indicate the study's design with a commonly used term in the title or the abstract	1
		**(b)** Provide in the abstract an informative and balanced summary of what was done and what was found	1
	**Introduction**		
**2**	*Background/Rationale*	Explain the scientific background and rationale for the investigation being reported	1-2
**3**	*Objectives*	State specific objectives, including any prespecified hypotheses	2
	**Methods**		
**4**	*Study design*	Present key elements of study design early in the paper	2
**5**	*Setting*	Describe the setting, locations, and relevant dates, including periods of recruitment, exposure, follow-up, and data collection	2
**6**	*Participants*	**(a)** Give the eligibility criteria, and the sources and methods of selection of participants	2-3

**7**	*Variables*	Clearly define all outcomes, exposures, predictors, potential confounders, and effect modifiers. Give diagnostic criteria, if applicable	3
**8**	*Data sources/ measurement*	For each variable of interest, give sources of data and details of methods of assessment (measurement). Describe comparability of assessment methods if there is more than one group	3
**9**	*Bias*	Describe any efforts to address potential sources of bias	
**10**	*Study size*	Explain how the study size was arrived at	3
**11**	*Quantitative variables*	Explain how quantitative variables were handled in the analyses. If applicable, describe which groupings were chosen and why	
**12**	*Statistical methods*	**(a)** Describe all statistical methods, including those used to control for confounding	4
		**(b)** Describe any methods used to examine subgroups and interactions	4
		**(c)** Explain how missing data were addressed	
		**(d)** If applicable, describe analytical methods taking account of sampling strategy	3
		**(e)** Describe any sensitivity analyses	
	**Results**		
**13**	*Participants*	**(a)** Report numbers of individuals at each stage of study—eg numbers potentially eligible, examined for eligibility, confirmed eligible, included in the study, completing follow-up, and analysed	4
		**(b)** Give reasons for non-participation at each stage	4
		**(c)** Consider use of a flow diagram	
**14**	*Descriptive data*	**(a)** Give characteristics of study participants (eg demographic, clinical, social) and information on exposures and potential confounders	4-5
		**(b)** Indicate number of participants with missing data for each variable of interest	
**15**	*Outcome data*	Report numbers of outcome events or summary measures	4-8
**16**	*Main results*	**(a)** Give unadjusted estimates and, if applicable, confounder-adjusted estimates and their precision (eg, 95% confidence interval). Make clear which confounders were adjusted for and why they were included	4-8
		**(b)** Report category boundaries when continuous variables were categorized	
		**(c)** If relevant, consider translating estimates of relative risk into absolute risk for a meaningful time period	
**17**	*Other analyses*	Report other analyses done—eg analyses of subgroups and interactions, and sensitivity analyses	
	**Discussion**		
**18**	*Key results*	Summarise key results with reference to study objectives	8
**19**	*Limitations*	Discuss limitations of the study, taking into account sources of potential bias or imprecision. Discuss both direction and magnitude of any potential bias	9
**20**	*Interpretation*	Give a cautious overall interpretation of results considering objectives, limitations, multiplicity of analyses, results from similar studies, and other relevant evidence	8-9
**21**	*Generalisability*	Discuss the generalisability (external validity) of the study results	9
	**Other information**		
**22**	Funding	Give the source of funding and the role of the funders for the present study and, if applicable, for the original study on which the present article is based	
**TOT.**			**18**

STROBE CHECKLIST ***CROSS-SECTIONAL STUDIES*** **- ARTICLE 3**

TITLE: Impact of school-based dental program performance on the oral health-related quality of life in children

Amalia et al. (2017)

Item		Recommendation	Page n°
1	**Title and Abstract**	**(a)** Indicate the study's design with a commonly used term in the title or the abstract	1
		**(b)** Provide in the abstract an informative and balanced summary of what was done and what was found	1
	**Introduction**		
2	*Background/Rationale*	Explain the scientific background and rationale for the investigation being reported	1-2
3	*Objectives*	State specific objectives, including any prespecified hypotheses	2
	**Methods**		
4	*Study design*	Present key elements of study design early in the paper	2
5	*Setting*	Describe the setting, locations, and relevant dates, including periods of recruitment, exposure, follow-up, and data collection	2
6	*Participants*	**(a)** Give the eligibility criteria, and the sources and methods of selection of participants	3
7	*Variables*	Clearly define all outcomes, exposures, predictors, potential confounders, and effect modifiers. Give diagnostic criteria, if applicable	2-4
8	*Data sources/ measurement*	For each variable of interest, give sources of data and details of methods of assessment (measurement). Describe comparability of assessment methods if there is more than one group	2-4
9	*Bias*	Describe any efforts to address potential sources of bias	
10	*Study size*	Explain how the study size was arrived at	
11	*Quantitative variables*	Explain how quantitative variables were handled in the analyses. If applicable, describe which groupings were chosen and why	
12	*Statistical methods*	**(a)** Describe all statistical methods, including those used to control for confounding	3-4
		**(b)** Describe any methods used to examine subgroups and interactions	
		**(c)** Explain how missing data were addressed	
		**(d)** If applicable, describe analytical methods taking account of sampling strategy	
		**(e)** Describe any sensitivity analyses	
	**Results**		
13	*Participants*	**(a)** Report numbers of individuals at each stage of study—eg numbers potentially eligible, examined for eligibility, confirmed eligible, included in the study, completing follow-up, and analysed	4
		**(b)** Give reasons for non-participation at each stage	
		**(c)** Consider use of a flow diagram	

**14**	*Descriptive data*	**(a)** Give characteristics of study participants (eg demographic, clinical, social) and information on exposures and potential confounders	4
		**(b)** Indicate number of participants with missing data for each variable of interest	
**15**	*Outcome data*	Report numbers of outcome events or summary measures	4-5
**16**	*Main results*	**(a)** Give unadjusted estimates and, if applicable, confounder-adjusted estimates and their precision (eg, 95% confidence interval). Make clear which confounders were adjusted for and why they were included	
		**(b)** Report category boundaries when continuous variables were categorized	
		**(c)** If relevant, consider translating estimates of relative risk into absolute risk for a meaningful time period	
**17**	*Other analyses*	Report other analyses done—eg analyses of subgroups and interactions, and sensitivity analyses	
	**Discussion**		
**18**	*Key results*	Summarise key results with reference to study objectives	5
**19**	*Limitations*	Discuss limitations of the study, taking into account sources of potential bias or imprecision. Discuss both direction and magnitude of any potential bias	6
**20**	*Interpretation*	Give a cautious overall interpretation of results considering objectives, limitations, multiplicity of analyses, results from similar studies, and other relevant evidence	6
**21**	*Generalisability*	Discuss the generalisability (external validity) of the study results	6
	**Other information**		
**22**	Funding	Give the source of funding and the role of the funders for the present study and, if applicable, for the original study on which the present article is based	
**TOT.**			16

STROBE CHECKLIST *CROSS-SECTIONAL STUDIES* - ARTICLE 4

TITLE: Oral Health-Related Quality of Life of School Children Aged 12-17 Years According to the Child-Oral Impacts on Daily Performances Index and the Impact of Oral Health Status on Index Scores

Athira et al. (2015)

Item		Recommendation	Page n°
1	**Title and Abstract**	**(a)** Indicate the study's design with a commonly used term in the title or the abstract	25
		**(b)** Provide in the abstract an informative and balanced summary of what was done and what was found	25
	**Introduction**		
2	*Background/Rationale*	Explain the scientific background and rationale for the investigation being reported	25-26
3	*Objectives*	State specific objectives, including any prespecified hypotheses	26
	**Methods**		

**4**	*Study design*	Present key elements of study design early in the paper	
**5**	*Setting*	Describe the setting, locations, and relevant dates, including periods of recruitment, exposure, follow-up, and data collection	26
**6**	*Participants*	**(a)** Give the eligibility criteria, and the sources and methods of selection of participants	26
**7**	*Variables*	Clearly define all outcomes, exposures, predictors, potential confounders, and effect modifiers. Give diagnostic criteria, if applicable	26-27
**8**	*Data sources/ measurement*	For each variable of interest, give sources of data and details of methods of assessment (measurement). Describe comparability of assessment methods if there is more than one group	27
**9**	*Bias*	Describe any efforts to address potential sources of bias	
**10**	*Study size*	Explain how the study size was arrived at	26
**11**	*Quantitative variables*	Explain how quantitative variables were handled in the analyses. If applicable, describe which groupings were chosen and why	
**12**	*Statistical methods*	**(a)** Describe all statistical methods, including those used to control for confounding	27
		**(b)** Describe any methods used to examine subgroups and interactions	
		**(c)** Explain how missing data were addressed	
		**(d)** If applicable, describe analytical methods taking account of sampling strategy	26
		**(e)** Describe any sensitivity analyses	
	**Results**		
**13**	*Participants*	**(a)** Report numbers of individuals at each stage of study—eg numbers potentially eligible, examined for eligibility, confirmed eligible, included in the study, completing follow-up, and analysed	27
		**(b)** Give reasons for non-participation at each stage	
		**(c)** Consider use of a flow diagram	
**14**	*Descriptive data*	**(a)** Give characteristics of study participants (eg demographic, clinical, social) and information on exposures and potential confounders	27-28
		**(b)** Indicate number of participants with missing data for each variable of interest	
**15**	*Outcome data*	Report numbers of outcome events or summary measures	
**16**	*Main results*	**(a)** Give unadjusted estimates and, if applicable, confounder-adjusted estimates and their precision (eg, 95% confidence interval). Make clear which confounders were adjusted for and why they were included	27-28
		**(b)** Report category boundaries when continuous variables were categorized	
		**(c)** If relevant, consider translating estimates of relative risk into absolute risk for a meaningful time period	
**17**	*Other analyses*	Report other analyses done—eg analyses of subgroups and interactions, and sensitivity analyses	
	**Discussion**		
**18**	*Key results*	Summarise key results with reference to study objectives	28
**19**	*Limitations*	Discuss limitations of the study, taking into account sources of potential bias or imprecision. Discuss both direction and magnitude of any potential bias	30
**20**	*Interpretation*	Give a cautious overall interpretation of results considering objectives, limitations, multiplicity of analyses, results from similar studies, and other relevant evidence	28-29
**21**	*Generalisability*	Discuss the generalisability (external validity) of the study results	28-29
	**Other information**		

22	Funding	Give the source of funding and the role of the funders for the present study and, if applicable, for the original study on which the present article is based	
**TOT.**			**16**

STROBE CHECKLIST *CROSS-SECTIONAL STUDIES* - ARTICLE 5

TITLE: Association of Oral Health Indicators with Quality-of-Life Related to Oral Health among Iranian Adolescent.

Bakhtiar et al. (2014)

Item		Recommendation	Page n°
1	**Title and Abstract**	**(a)** Indicate the study's design with a commonly used term in the title or the abstract	5
		**(b)** Provide in the abstract an informative and balanced summary of what was done and what was found	5
	**Introduction**		
2	*Background/Rationale*	Explain the scientific background and rationale for the investigation being reported	5
3	*Objectives*	State specific objectives, including any prespecified hypotheses	5-6
	**Methods**		
4	*Study design*	Present key elements of study design early in the paper	6
5	*Setting*	Describe the setting, locations, and relevant dates, including periods of recruitment, exposure, follow-up, and data collection	6
6	*Participants*	**(a)** Give the eligibility criteria, and the sources and methods of selection of participants	6
7	*Variables*	Clearly define all outcomes, exposures, predictors, potential confounders, and effect modifiers. Give diagnostic criteria, if applicable	6
8	*Data sources/ measurement*	For each variable of interest, give sources of data and details of methods of assessment (measurement). Describe comparability of assessment methods if there is more than one group	6
9	*Bias*	Describe any efforts to address potential sources of bias	
10	*Study size*	Explain how the study size was arrived at	
11	*Quantitative variables*	Explain how quantitative variables were handled in the analyses. If applicable, describe which groupings were chosen and why	
12	*Statistical methods*	**(a)** Describe all statistical methods, including those used to control for confounding	6
		**(b)** Describe any methods used to examine subgroups and interactions	
		**(c)** Explain how missing data were addressed	
		**(d)** If applicable, describe analytical methods taking account of sampling strategy	6
		**(e)** Describe any sensitivity analyses	
	**Results**		
13	*Participants*	**(a)** Report numbers of individuals at each stage of study—eg numbers potentially eligible, examined for eligibility, confirmed eligible, included in the study, completing follow-up, and analysed	6

		**(b)** Give reasons for non-participation at each stage	
		**(c)** Consider use of a flow diagram	
**14**	*Descriptive data*	**(a)** Give characteristics of study participants (eg demographic, clinical, social) and information on exposures and potential confounders	6
		**(b)** Indicate number of participants with missing data for each variable of interest	
**15**	*Outcome data*	Report numbers of outcome events or summary measures	6-7
**16**	*Main results*	**(a)** Give unadjusted estimates and, if applicable, confounder-adjusted estimates and their precision (eg, 95% confidence interval). Make clear which confounders were adjusted for and why they were included	6-7
		**(b)** Report category boundaries when continuous variables were categorized	
		**(c)** If relevant, consider translating estimates of relative risk into absolute risk for a meaningful time period	
**17**	*Other analyses*	Report other analyses done—eg analyses of subgroups and interactions, and sensitivity analyses	
	**Discussion**		
**18**	*Key results*	Summarise key results with reference to study objectives	7
**19**	*Limitations*	Discuss limitations of the study, taking into account sources of potential bias or imprecision. Discuss both direction and magnitude of any potential bias	
**20**	*Interpretation*	Give a cautious overall interpretation of results considering objectives, limitations, multiplicity of analyses, results from similar studies, and other relevant evidence	7-8
**21**	*Generalisability*	Discuss the generalisability (external validity) of the study results	7-8
	**Other information**		
**22**	Funding	Give the source of funding and the role of the funders for the present study and, if applicable, for the original study on which the present article is based	
**TOT.**			**16**

STROBE CHECKLIST *CROSS-SECTIONAL STUDIES* - ARTICLE 6

TITLE: Correlation Between Oral Health and Child-OIDP Index in 12-and 15-Year-Old Children From Modinagar, India

Basavaraj et al. (2014)

**Item**		**Recommendation**	**Page n°**
**1**	**Title and Abstract**	**(a)** Indicate the study's design with a commonly used term in the title or the abstract	1
		**(b)** Provide in the abstract an informative and balanced summary of what was done and what was found	1
	**Introduction**		
**2**	*Background/Rationale*	Explain the scientific background and rationale for the investigation being reported	1-2
**3**	*Objectives*	State specific objectives, including any prespecified hypotheses	2

	**Methods**		
**4**	*Study design*	Present key elements of study design early in the paper	2
**5**	*Setting*	Describe the setting, locations, and relevant dates, including periods of recruitment, exposure, follow-up, and data collection	2-3
**6**	*Participants*	**(a)** Give the eligibility criteria, and the sources and methods of selection of participants	2-3
**7**	*Variables*	Clearly define all outcomes, exposures, predictors, potential confounders, and effect modifiers. Give diagnostic criteria, if applicable	3-4
**8**	*Data sources/ measurement*	For each variable of interest, give sources of data and details of methods of assessment (measurement). Describe comparability of assessment methods if there is more than one group	4
**9**	*Bias*	Describe any efforts to address potential sources of bias	3
**10**	*Study size*	Explain how the study size was arrived at	3
**11**	*Quantitative variables*	Explain how quantitative variables were handled in the analyses. If applicable, describe which groupings were chosen and why	
**12**	*Statistical methods*	**(a)** Describe all statistical methods, including those used to control for confounding	4-5
		**(b)** Describe any methods used to examine subgroups and interactions	5
		**(c)** Explain how missing data were addressed	
		**(d)** If applicable, describe analytical methods taking account of sampling strategy	
		**(e)** Describe any sensitivity analyses	
	**Results**		
**13**	*Participants*	**(a)** Report numbers of individuals at each stage of study—eg numbers potentially eligible, examined for eligibility, confirmed eligible, included in the study, completing follow-up, and analysed	5
		**(b)** Give reasons for non-participation at each stage	
		**(c)** Consider use of a flow diagram	
**14**	*Descriptive data*	**(a)** Give characteristics of study participants (eg demographic, clinical, social) and information on exposures and potential confounders	5-7
		**(b)** Indicate number of participants with missing data for each variable of interest	
**15**	*Outcome data*	Report numbers of outcome events or summary measures	5-7
**16**	*Main results*	**(a)** Give unadjusted estimates and, if applicable, confounder-adjusted estimates and their precision (eg, 95% confidence interval). Make clear which confounders were adjusted for and why they were included	5-7
		**(b)** Report category boundaries when continuous variables were categorized	
		**(c)** If relevant, consider translating estimates of relative risk into absolute risk for a meaningful time period	
**17**	*Other analyses*	Report other analyses done—eg analyses of subgroups and interactions, and sensitivity analyses	
	**Discussion**		
**18**	*Key results*	Summarise key results with reference to study objectives	8
**19**	*Limitations*	Discuss limitations of the study, taking into account sources of potential bias or imprecision. Discuss both direction and magnitude of any potential bias	9
**20**	*Interpretation*	Give a cautious overall interpretation of results considering objectives, limitations, multiplicity of analyses, results from similar studies, and other relevant evidence	8-9
**21**	*Generalisability*	Discuss the generalisability (external validity) of the study results	9

	**Other information**		
**22**	Funding	Give the source of funding and the role of the funders for the present study and, if applicable, for the original study on which the present article is based	9
**TOT.**			20

STROBE CHECKLIST *CROSS-SECTIONAL STUDIES* - ARTICLE 7

TITLE: Intensity and extent of oral impacts on daily performances by type of self-perceived oral problems

Bernabé et al. (2007)

**Item**		**Recommendation**	**Page n°**
**1**	**Title and Abstract**	**(a)** Indicate the study's design with a commonly used term in the title or the abstract	
		**(b)** Provide in the abstract an informative and balanced summary of what was done and what was found	111
	**Introduction**		
**2**	*Background/Rationale*	Explain the scientific background and rationale for the investigation being reported	111
**3**	*Objectives*	State specific objectives, including any prespecified hypotheses	111
	**Methods**		
**4**	*Study design*	Present key elements of study design early in the paper	
**5**	*Setting*	Describe the setting, locations, and relevant dates, including periods of recruitment, exposure, follow-up, and data collection	111-112
**6**	*Participants*	**(a)** Give the eligibility criteria, and the sources and methods of selection of participants	111-112
**7**	*Variables*	Clearly define all outcomes, exposures, predictors, potential confounders, and effect modifiers. Give diagnostic criteria, if applicable	111-112
**8**	*Data sources/ measurement*	For each variable of interest, give sources of data and details of methods of assessment (measurement). Describe comparability of assessment methods if there is more than one group	111-112
**9**	*Bias*	Describe any efforts to address potential sources of bias	
**10**	*Study size*	Explain how the study size was arrived at	111
**11**	*Quantitative variables*	Explain how quantitative variables were handled in the analyses. If applicable, describe which groupings were chosen and why	
**12**	*Statistical methods*	**(a)** Describe all statistical methods, including those used to control for confounding	112
		**(b)** Describe any methods used to examine subgroups and interactions	112
		**(c)** Explain how missing data were addressed	
		**(d)** If applicable, describe analytical methods taking account of sampling strategy	
		**(e)** Describe any sensitivity analyses	
	**Results**		
**13**	*Participants*	**(a)** Report numbers of individuals at each stage of study—eg numbers potentially eligible, examined for eligibility, confirmed eligible, included in the study, completing follow-up, and analysed	112

		**(b)** Give reasons for non-participation at each stage	
		**(c)** Consider use of a flow diagram	
**14**	*Descriptive data*	**(a)** Give characteristics of study participants (eg demographic, clinical, social) and information on exposures and potential confounders	
		**(b)** Indicate number of participants with missing data for each variable of interest	
**15**	*Outcome data*	Report numbers of outcome events or summary measures	112-114
**16**	*Main results*	**(a)** Give unadjusted estimates and, if applicable, confounder-adjusted estimates and their precision (eg, 95% confidence interval). Make clear which confounders were adjusted for and why they were included	112-114
		**(b)** Report category boundaries when continuous variables were categorized	
		**(c)** If relevant, consider translating estimates of relative risk into absolute risk for a meaningful time period	
**17**	*Other analyses*	Report other analyses done—eg analyses of subgroups and interactions, and sensitivity analyses	
	**Discussion**		
**18**	*Key results*	Summarise key results with reference to study objectives	114
**19**	*Limitations*	Discuss limitations of the study, taking into account sources of potential bias or imprecision. Discuss both direction and magnitude of any potential bias	
**20**	*Interpretation*	Give a cautious overall interpretation of results considering objectives, limitations, multiplicity of analyses, results from similar studies, and other relevant evidence	114-115
**21**	*Generalisability*	Discuss the generalisability (external validity) of the study results	115
	**Other information**		
**22**	Funding	Give the source of funding and the role of the funders for the present study and, if applicable, for the original study on which the present article is based	
**TOT.**			15

STROBE CHECKLIST *CROSS-SECTIONAL STUDIES* - ARTICLE 8

TITLE: Prevalence and determinants of oral impacts on daily performance: results from a survey among school children in Italy

Bianco et al. (2009)

Item		Recommendation	Page n°
**1**	**Title and Abstract**	**(a)** Indicate the study's design with a commonly used term in the title or the abstract	
		**(b)** Provide in the abstract an informative and balanced summary of what was done and what was found	595
	**Introduction**		
**2**	*Background/Rationale*	Explain the scientific background and rationale for the investigation being reported	595
**3**	*Objectives*	State specific objectives, including any prespecified hypotheses	595
	**Methods**		
**4**	*Study design*	Present key elements of study design early in the paper	595

**5**	*Setting*	Describe the setting, locations, and relevant dates, including periods of recruitment, exposure, follow-up, and data collection	595
**6**	*Participants*	**(a)** Give the eligibility criteria, and the sources and methods of selection of participants	595
**7**	*Variables*	Clearly define all outcomes, exposures, predictors, potential confounders, and effect modifiers. Give diagnostic criteria, if applicable	595-596
**8**	*Data sources/ measurement*	For each variable of interest, give sources of data and details of methods of assessment (measurement). Describe comparability of assessment methods if there is more than one group	595-596
**9**	*Bias*	Describe any efforts to address potential sources of bias	
**10**	*Study size*	Explain how the study size was arrived at	595
**11**	*Quantitative variables*	Explain how quantitative variables were handled in the analyses. If applicable, describe which groupings were chosen and why	
**12**	*Statistical methods*	**(a)** Describe all statistical methods, including those used to control for confounding	596
		**(b)** Describe any methods used to examine subgroups and interactions	
		**(c)** Explain how missing data were addressed	
		**(d)** If applicable, describe analytical methods taking account of sampling strategy	
		**(e)** Describe any sensitivity analyses	
	**Results**		
**13**	*Participants*	**(a)** Report numbers of individuals at each stage of study—eg numbers potentially eligible, examined for eligibility, confirmed eligible, included in the study, completing follow-up, and analysed	596
		**(b)** Give reasons for non-participation at each stage	
		**(c)** Consider use of a flow diagram	
**14**	*Descriptive data*	**(a)** Give characteristics of study participants (eg demographic, clinical, social) and information on exposures and potential confounders	596-598
		**(b)** Indicate number of participants with missing data for each variable of interest	
**15**	*Outcome data*	Report numbers of outcome events or summary measures	596-598
**16**	*Main results*	**(a)** Give unadjusted estimates and, if applicable, confounder-adjusted estimates and their precision (eg, 95% confidence interval). Make clear which confounders were adjusted for and why they were included	596-598
		**(b)** Report category boundaries when continuous variables were categorized	
		**(c)** If relevant, consider translating estimates of relative risk into absolute risk for a meaningful time period	
**17**	*Other analyses*	Report other analyses done—eg analyses of subgroups and interactions, and sensitivity analyses	
	**Discussion**		
**18**	*Key results*	Summarise key results with reference to study objectives	598
**19**	*Limitations*	Discuss limitations of the study, taking into account sources of potential bias or imprecision. Discuss both direction and magnitude of any potential bias	599
**20**	*Interpretation*	Give a cautious overall interpretation of results considering objectives, limitations, multiplicity of analyses, results from similar studies, and other relevant evidence	598-599
**21**	*Generalisability*	Discuss the generalisability (external validity) of the study results	599
	**Other information**		

22	Funding	Give the source of funding and the role of the funders for the present study and, if applicable, for the original study on which the present article is based	
**TOT.**			18

STROBE CHECKLIST *CROSS-SECTIONAL STUDIES* - ARTICLE 9

TITLE: Oral health-related quality of life of 11- and 12-year-old public school children in Rio de Janeiro

Castro et al. (2011)

**Item**		**Recommendation**	**Page n°**
**1**	**Title and Abstract**	**(a)** Indicate the study's design with a commonly used term in the title or the abstract	336
		**(b)** Provide in the abstract an informative and balanced summary of what was done and what was found	336
	**Introduction**		
**2**	*Background/Rationale*	Explain the scientific background and rationale for the investigation being reported	336-337
**3**	*Objectives*	State specific objectives, including any prespecified hypotheses	337
	**Methods**		
**4**	*Study design*	Present key elements of study design early in the paper	337
**5**	*Setting*	Describe the setting, locations, and relevant dates, including periods of recruitment, exposure, follow-up, and data collection	337
**6**	*Participants*	**(a)** Give the eligibility criteria, and the sources and methods of selection of participants	337
**7**	*Variables*	Clearly define all outcomes, exposures, predictors, potential confounders, and effect modifiers. Give diagnostic criteria, if applicable	337-338
**8**	*Data sources/ measurement*	For each variable of interest, give sources of data and details of methods of assessment (measurement). Describe comparability of assessment methods if there is more than one group	337-338
**9**	*Bias*	Describe any efforts to address potential sources of bias	
**10**	*Study size*	Explain how the study size was arrived at	
**11**	*Quantitative variables*	Explain how quantitative variables were handled in the analyses. If applicable, describe which groupings were chosen and why	
**12**	*Statistical methods*	**(a)** Describe all statistical methods, including those used to control for confounding	338-339
		**(b)** Describe any methods used to examine subgroups and interactions	338-339
		**(c)** Explain how missing data were addressed	
		**(d)** If applicable, describe analytical methods taking account of sampling strategy	
		**(e)** Describe any sensitivity analyses	
	**Results**		
**13**	*Participants*	**(a)** Report numbers of individuals at each stage of study—eg numbers potentially eligible, examined for eligibility, confirmed eligible, included in the study, completing follow-up, and analysed	339
		**(b)** Give reasons for non-participation at each stage	339

		**(c)** Consider use of a flow diagram	
**14**	*Descriptive data*	**(a)** Give characteristics of study participants (eg demographic, clinical, social) and information on exposures and potential confounders	339-341
		**(b)** Indicate number of participants with missing data for each variable of interest	
**15**	*Outcome data*	Report numbers of outcome events or summary measures	339-341
**16**	*Main results*	**(a)** Give unadjusted estimates and, if applicable, confounder-adjusted estimates and their precision (eg, 95% confidence interval). Make clear which confounders were adjusted for and why they were included	339-341
		**(b)** Report category boundaries when continuous variables were categorized	
		**(c)** If relevant, consider translating estimates of relative risk into absolute risk for a meaningful time period	
**17**	*Other analyses*	Report other analyses done—eg analyses of subgroups and interactions, and sensitivity analyses	
	**Discussion**		
**18**	*Key results*	Summarise key results with reference to study objectives	341
**19**	*Limitations*	Discuss limitations of the study, taking into account sources of potential bias or imprecision. Discuss both direction and magnitude of any potential bias	341
**20**	*Interpretation*	Give a cautious overall interpretation of results considering objectives, limitations, multiplicity of analyses, results from similar studies, and other relevant evidence	342-343
**21**	*Generalisability*	Discuss the generalisability (external validity) of the study results	343
	**Other information**		
**22**	Funding	Give the source of funding and the role of the funders for the present study and, if applicable, for the original study on which the present article is based	
**TOT.**			**16**

STROBE CHECKLIST *CROSS-SECTIONAL STUDIES* - ARTICLE 10

TITLE: Impact of oral conditions on the quality of life in rural schoolchildren, Piura, Peru

Del Castillo-López et al. (2014)

**Item**		**Recommendation**	**Page n°**
**1**	**Title and Abstract**	**(a)** Indicate the study's design with a commonly used term in the title or the abstract	220
		**(b)** Provide in the abstract an informative and balanced summary of what was done and what was found	220-221
	**Introduction**		
**2**	*Background/Rationale*	Explain the scientific background and rationale for the investigation being reported	221-222
**3**	*Objectives*	State specific objectives, including any prespecified hypotheses	222
	**Methods**		
**4**	*Study design*	Present key elements of study design early in the paper	222

**5**	*Setting*	Describe the setting, locations, and relevant dates, including periods of recruitment, exposure, follow-up, and data collection	222
**6**	*Participants*	**(a)** Give the eligibility criteria, and the sources and methods of selection of participants	
**7**	*Variables*	Clearly define all outcomes, exposures, predictors, potential confounders, and effect modifiers. Give diagnostic criteria, if applicable	222
**8**	*Data sources/ measurement*	For each variable of interest, give sources of data and details of methods of assessment (measurement). Describe comparability of assessment methods if there is more than one group	222
**9**	*Bias*	Describe any efforts to address potential sources of bias	
**10**	*Study size*	Explain how the study size was arrived at	222
**11**	*Quantitative variables*	Explain how quantitative variables were handled in the analyses. If applicable, describe which groupings were chosen and why	
**12**	*Statistical methods*	**(a)** Describe all statistical methods, including those used to control for confounding	222-223
		**(b)** Describe any methods used to examine subgroups and interactions	
		**(c)** Explain how missing data were addressed	
		**(d)** If applicable, describe analytical methods taking account of sampling strategy	
		**(e)** Describe any sensitivity analyses	
	**Results**		
**13**	*Participants*	**(a)** Report numbers of individuals at each stage of study—eg numbers potentially eligible, examined for eligibility, confirmed eligible, included in the study, completing follow-up, and analysed	223
		**(b)** Give reasons for non-participation at each stage	223
		**(c)** Consider use of a flow diagram	
**14**	*Descriptive data*	**(a)** Give characteristics of study participants (eg demographic, clinical, social) and information on exposures and potential confounders	223-225
		**(b)** Indicate number of participants with missing data for each variable of interest	
**15**	*Outcome data*	Report numbers of outcome events or summary measures	223-226
**16**	*Main results*	**(a)** Give unadjusted estimates and, if applicable, confounder-adjusted estimates and their precision (eg, 95% confidence interval). Make clear which confounders were adjusted for and why they were included	223-226
		**(b)** Report category boundaries when continuous variables were categorized	
		**(c)** If relevant, consider translating estimates of relative risk into absolute risk for a meaningful time period	
**17**	*Other analyses*	Report other analyses done—eg analyses of subgroups and interactions, and sensitivity analyses	
	**Discussion**		
**18**	*Key results*	Summarise key results with reference to study objectives	226
**19**	*Limitations*	Discuss limitations of the study, taking into account sources of potential bias or imprecision. Discuss both direction and magnitude of any potential bias	227
**20**	*Interpretation*	Give a cautious overall interpretation of results considering objectives, limitations, multiplicity of analyses, results from similar studies, and other relevant evidence	226-227
**21**	*Generalisability*	Discuss the generalisability (external validity) of the study results	227
	**Other information**		

22	Funding	Give the source of funding and the role of the funders for the present study and, if applicable, for the original study on which the present article is based	
**TOT.**			17

STROBE CHECKLIST *CROSS-SECTIONAL STUDIES* - ARTICLE 11

TITLE: Oral Impacts on Daily Performances of Children 12 and 15-Year-Old in Can Tho City

Do et al. (2020)

Item		Recommendation	Page n°
1	**Title and Abstract**	**(a)** Indicate the study's design with a commonly used term in the title or the abstract	
		**(b)** Provide in the abstract an informative and balanced summary of what was done and what was found	585
	**Introduction**		
2	*Background/Rationale*	Explain the scientific background and rationale for the investigation being reported	585
3	*Objectives*	State specific objectives, including any prespecified hypotheses	585
	**Methods**		
4	*Study design*	Present key elements of study design early in the paper	586
5	*Setting*	Describe the setting, locations, and relevant dates, including periods of recruitment, exposure, follow-up, and data collection	586
6	*Participants*	**(a)** Give the eligibility criteria, and the sources and methods of selection of participants	586
7	*Variables*	Clearly define all outcomes, exposures, predictors, potential confounders, and effect modifiers. Give diagnostic criteria, if applicable	586
8	*Data sources/ measurement*	For each variable of interest, give sources of data and details of methods of assessment (measurement). Describe comparability of assessment methods if there is more than one group	586
9	*Bias*	Describe any efforts to address potential sources of bias	
10	*Study size*	Explain how the study size was arrived at	586
11	*Quantitative variables*	Explain how quantitative variables were handled in the analyses. If applicable, describe which groupings were chosen and why	
12	*Statistical methods*	**(a)** Describe all statistical methods, including those used to control for confounding	586
		**(b)** Describe any methods used to examine subgroups and interactions	
		**(c)** Explain how missing data were addressed	
		**(d)** If applicable, describe analytical methods taking account of sampling strategy	586
		**(e)** Describe any sensitivity analyses	
	**Results**		
13	*Participants*	**(a)** Report numbers of individuals at each stage of study—eg numbers potentially eligible, examined for eligibility, confirmed eligible, included in the study, completing follow-up, and analysed	586
		**(b)** Give reasons for non-participation at each stage	
		**(c)** Consider use of a flow diagram	

**14**	*Descriptive data*	**(a)** Give characteristics of study participants (eg demographic, clinical, social) and information on exposures and potential confounders	586-587
		**(b)** Indicate number of participants with missing data for each variable of interest	
**15**	*Outcome data*	Report numbers of outcome events or summary measures	586-588
**16**	*Main results*	**(a)** Give unadjusted estimates and, if applicable, confounder-adjusted estimates and their precision (eg, 95% confidence interval). Make clear which confounders were adjusted for and why they were included	586-588
		**(b)** Report category boundaries when continuous variables were categorized	
		**(c)** If relevant, consider translating estimates of relative risk into absolute risk for a meaningful time period	
**17**	*Other analyses*	Report other analyses done—eg analyses of subgroups and interactions, and sensitivity analyses	
	**Discussion**		
**18**	*Key results*	Summarise key results with reference to study objectives	588
**19**	*Limitations*	Discuss limitations of the study, taking into account sources of potential bias or imprecision. Discuss both direction and magnitude of any potential bias	
**20**	*Interpretation*	Give a cautious overall interpretation of results considering objectives, limitations, multiplicity of analyses, results from similar studies, and other relevant evidence	588-589
**21**	*Generalisability*	Discuss the generalisability (external validity) of the study results	588-589
	**Other information**		
**22**	Funding	Give the source of funding and the role of the funders for the present study and, if applicable, for the original study on which the present article is based	
**TOT.**			17

STROBE CHECKLIST *CROSS-SECTIONAL STUDIES* - <u>ARTICLE 12</u>

TITLE: Life quality related to oral health of schoolchildren from Bucharest

Dumitrache et al. (2009)

Item		Recommendation	Page n°
**1**	**Title and Abstract**	**(a)** Indicate the study's design with a commonly used term in the title or the abstract	169
		**(b)** Provide in the abstract an informative and balanced summary of what was done and what was found	169
	**Introduction**		
**2**	*Background/Rationale*	Explain the scientific background and rationale for the investigation being reported	170-171
**3**	*Objectives*	State specific objectives, including any prespecified hypotheses	
	**Methods**		
**4**	*Study design*	Present key elements of study design early in the paper	171

**5**	*Setting*	Describe the setting, locations, and relevant dates, including periods of recruitment, exposure, follow-up, and data collection	171
**6**	*Participants*	**(a)** Give the eligibility criteria, and the sources and methods of selection of participants	171
**7**	*Variables*	Clearly define all outcomes, exposures, predictors, potential confounders, and effect modifiers. Give diagnostic criteria, if applicable	171-172
**8**	*Data sources/ measurement*	For each variable of interest, give sources of data and details of methods of assessment (measurement). Describe comparability of assessment methods if there is more than one group	171-172
**9**	*Bias*	Describe any efforts to address potential sources of bias	
**10**	*Study size*	Explain how the study size was arrived at	
**11**	*Quantitative variables*	Explain how quantitative variables were handled in the analyses. If applicable, describe which groupings were chosen and why	
**12**	*Statistical methods*	**(a)** Describe all statistical methods, including those used to control for confounding	172
		**(b)** Describe any methods used to examine subgroups and interactions	
		**(c)** Explain how missing data were addressed	
		**(d)** If applicable, describe analytical methods taking account of sampling strategy	
		**(e)** Describe any sensitivity analyses	
	**Results**		
**13**	*Participants*	**(a)** Report numbers of individuals at each stage of study—eg numbers potentially eligible, examined for eligibility, confirmed eligible, included in the study, completing follow-up, and analysed	173
		**(b)** Give reasons for non-participation at each stage	173
		**(c)** Consider use of a flow diagram	
**14**	*Descriptive data*	**(a)** Give characteristics of study participants (eg demographic, clinical, social) and information on exposures and potential confounders	173-175
		**(b)** Indicate number of participants with missing data for each variable of interest	
**15**	*Outcome data*	Report numbers of outcome events or summary measures	173-175
**16**	*Main results*	**(a)** Give unadjusted estimates and, if applicable, confounder-adjusted estimates and their precision (eg, 95% confidence interval). Make clear which confounders were adjusted for and why they were included	173-175
		**(b)** Report category boundaries when continuous variables were categorized	
		**(c)** If relevant, consider translating estimates of relative risk into absolute risk for a meaningful time period	
**17**	*Other analyses*	Report other analyses done—eg analyses of subgroups and interactions, and sensitivity analyses	
	**Discussion**		
**18**	*Key results*	Summarise key results with reference to study objectives	175
**19**	*Limitations*	Discuss limitations of the study, taking into account sources of potential bias or imprecision. Discuss both direction and magnitude of any potential bias	
**20**	*Interpretation*	Give a cautious overall interpretation of results considering objectives, limitations, multiplicity of analyses, results from similar studies, and other relevant evidence	175-176
**21**	*Generalisability*	Discuss the generalisability (external validity) of the study results	
	**Other information**		

**22**	Funding	Give the source of funding and the role of the funders for the present study and, if applicable, for the original study on which the present article is based	
**TOT.**			**14**

STROBE CHECKLIST *CROSS-SECTIONAL STUDIES* - ARTICLE 13

TITLE: Oral health impact, dental caries experience, and associated factors in 12-15-year-old school children in India

Kumar et al. (2015)

**Item**		**Recommendation**	**Page n°**
**1**	**Title and Abstract**	**(a)** Indicate the study's design with a commonly used term in the title or the abstract	1
		**(b)** Provide in the abstract an informative and balanced summary of what was done and what was found	1
	**Introduction**		
**2**	*Background/Rationale*	Explain the scientific background and rationale for the investigation being reported	1-2
**3**	*Objectives*	State specific objectives, including any prespecified hypotheses	2
	**Methods**		
**4**	*Study design*	Present key elements of study design early in the paper	2
**5**	*Setting*	Describe the setting, locations, and relevant dates, including periods of recruitment, exposure, follow-up, and data collection	2
**6**	*Participants*	**(a)** Give the eligibility criteria, and the sources and methods of selection of participants	2
**7**	*Variables*	Clearly define all outcomes, exposures, predictors, potential confounders, and effect modifiers. Give diagnostic criteria, if applicable	2
**8**	*Data sources/ measurement*	For each variable of interest, give sources of data and details of methods of assessment (measurement). Describe comparability of assessment methods if there is more than one group	2
**9**	*Bias*	Describe any efforts to address potential sources of bias	
**10**	*Study size*	Explain how the study size was arrived at	
**11**	*Quantitative variables*	Explain how quantitative variables were handled in the analyses. If applicable, describe which groupings were chosen and why	
**12**	*Statistical methods*	**(a)** Describe all statistical methods, including those used to control for confounding	2
		**(b)** Describe any methods used to examine subgroups and interactions	
		**(c)** Explain how missing data were addressed	
		**(d)** If applicable, describe analytical methods taking account of sampling strategy	
		**(e)** Describe any sensitivity analyses	
	**Results**		
**13**	*Participants*	**(a)** Report numbers of individuals at each stage of study—eg numbers potentially eligible, examined for eligibility, confirmed eligible, included in the study, completing follow-up, and analysed	2
		**(b)** Give reasons for non-participation at each stage	

		**(c)** Consider use of a flow diagram	
**14**	*Descriptive data*	**(a)** Give characteristics of study participants (eg demographic, clinical, social) and information on exposures and potential confounders	2-4
		**(b)** Indicate number of participants with missing data for each variable of interest	
**15**	*Outcome data*	Report numbers of outcome events or summary measures	2-4
**16**	*Main results*	**(a)** Give unadjusted estimates and, if applicable, confounder-adjusted estimates and their precision (eg, 95% confidence interval). Make clear which confounders were adjusted for and why they were included	2-4
		**(b)** Report category boundaries when continuous variables were categorized	
		**(c)** If relevant, consider translating estimates of relative risk into absolute risk for a meaningful time period	
**17**	*Other analyses*	Report other analyses done—eg analyses of subgroups and interactions, and sensitivity analyses	
	**Discussion**		
**18**	*Key results*	Summarise key results with reference to study objectives	3
**19**	*Limitations*	Discuss limitations of the study, taking into account sources of potential bias or imprecision. Discuss both direction and magnitude of any potential bias	5
**20**	*Interpretation*	Give a cautious overall interpretation of results considering objectives, limitations, multiplicity of analyses, results from similar studies, and other relevant evidence	3-5
**21**	*Generalisability*	Discuss the generalisability (external validity) of the study results	5
	**Other information**		
**22**	Funding	Give the source of funding and the role of the funders for the present study and, if applicable, for the original study on which the present article is based	
**TOT.**			17

STROBE CHECKLIST *CROSS-SECTIONAL STUDIES* - ARTICLE 14

TITLE: Oral Health Related Quality of Life in school children of urban-marginal area

Marcelo-Ingunza et al. (2015)

Item		Recommendation	Page n°
**1**	**Title and Abstract**	**(a)** Indicate the study's design with a commonly used term in the title or the abstract	194
		**(b)** Provide in the abstract an informative and balanced summary of what was done and what was found	194-195
	**Introduction**		
**2**	*Background/Rationale*	Explain the scientific background and rationale for the investigation being reported	195
**3**	*Objectives*	State specific objectives, including any prespecified hypotheses	195
	**Methods**		
**4**	*Study design*	Present key elements of study design early in the paper	195-196
**5**	*Setting*	Describe the setting, locations, and relevant dates, including periods of recruitment, exposure, follow-up, and data collection	195-196

**6**	*Participants*	**(a)** Give the eligibility criteria, and the sources and methods of selection of participants	195-196
**7**	*Variables*	Clearly define all outcomes, exposures, predictors, potential confounders, and effect modifiers. Give diagnostic criteria, if applicable	196
**8**	*Data sources/ measurement*	For each variable of interest, give sources of data and details of methods of assessment (measurement). Describe comparability of assessment methods if there is more than one group	195-196
**9**	*Bias*	Describe any efforts to address potential sources of bias	
**10**	*Study size*	Explain how the study size was arrived at	195-196
**11**	*Quantitative variables*	Explain how quantitative variables were handled in the analyses. If applicable, describe which groupings were chosen and why	
**12**	*Statistical methods*	**(a)** Describe all statistical methods, including those used to control for confounding	196
		**(b)** Describe any methods used to examine subgroups and interactions	
		**(c)** Explain how missing data were addressed	
		**(d)** If applicable, describe analytical methods taking account of sampling strategy	
		**(e)** Describe any sensitivity analyses	
	**Results**		
**13**	*Participants*	**(a)** Report numbers of individuals at each stage of study—eg numbers potentially eligible, examined for eligibility, confirmed eligible, included in the study, completing follow-up, and analysed	
		**(b)** Give reasons for non-participation at each stage	
		**(c)** Consider use of a flow diagram	
**14**	*Descriptive data*	**(a)** Give characteristics of study participants (eg demographic, clinical, social) and information on exposures and potential confounders	196-197
		**(b)** Indicate number of participants with missing data for each variable of interest	
**15**	*Outcome data*	Report numbers of outcome events or summary measures	196-197
**16**	*Main results*	**(a)** Give unadjusted estimates and, if applicable, confounder-adjusted estimates and their precision (eg, 95% confidence interval). Make clear which confounders were adjusted for and why they were included	196-197
		**(b)** Report category boundaries when continuous variables were categorized	
		**(c)** If relevant, consider translating estimates of relative risk into absolute risk for a meaningful time period	
**17**	*Other analyses*	Report other analyses done—eg analyses of subgroups and interactions, and sensitivity analyses	
	**Discussion**		
**18**	*Key results*	Summarise key results with reference to study objectives	197
**19**	*Limitations*	Discuss limitations of the study, taking into account sources of potential bias or imprecision. Discuss both direction and magnitude of any potential bias	203
**20**	*Interpretation*	Give a cautious overall interpretation of results considering objectives, limitations, multiplicity of analyses, results from similar studies, and other relevant evidence	197, 201-202
**21**	*Generalisability*	Discuss the generalisability (external validity) of the study results	203
	**Other information**		
**22**	Funding	Give the source of funding and the role of the funders for the present study and, if applicable, for the original study on which the present article is based	
**TOT.**			17

STROBE CHECKLIST *CROSS-SECTIONAL STUDIES* - ARTICLE 15

TITLE: Oral health impact on the quality of life of 11 to 14 years-old schoolchildren, Licantén, 2013

Moreno Ruiz et al. (2014)

Item		Recommendation	Page n°
1	**Title and Abstract**	**(a)** Indicate the study's design with a commonly used term in the title or the abstract	
		**(b)** Provide in the abstract an informative and balanced summary of what was done and what was found	142-143
	**Introduction**		
2	*Background/Rationale*	Explain the scientific background and rationale for the investigation being reported	143
3	*Objectives*	State specific objectives, including any prespecified hypotheses	143
	**Methods**		
4	*Study design*	Present key elements of study design early in the paper	
5	*Setting*	Describe the setting, locations, and relevant dates, including periods of recruitment, exposure, follow-up, and data collection	144
6	*Participants*	**(a)** Give the eligibility criteria, and the sources and methods of selection of participants	144
7	*Variables*	Clearly define all outcomes, exposures, predictors, potential confounders, and effect modifiers. Give diagnostic criteria, if applicable	144
8	*Data sources/ measurement*	For each variable of interest, give sources of data and details of methods of assessment (measurement). Describe comparability of assessment methods if there is more than one group	144
9	*Bias*	Describe any efforts to address potential sources of bias	
10	*Study size*	Explain how the study size was arrived at	
11	*Quantitative variables*	Explain how quantitative variables were handled in the analyses. If applicable, describe which groupings were chosen and why	
12	*Statistical methods*	**(a)** Describe all statistical methods, including those used to control for confounding	144
		**(b)** Describe any methods used to examine subgroups and interactions	
		**(c)** Explain how missing data were addressed	
		**(d)** If applicable, describe analytical methods taking account of sampling strategy	
		**(e)** Describe any sensitivity analyses	
	**Results**		
13	*Participants*	**(a)** Report numbers of individuals at each stage of study—eg numbers potentially eligible, examined for eligibility, confirmed eligible, included in the study, completing follow-up, and analysed	144
		**(b)** Give reasons for non-participation at each stage	
		**(c)** Consider use of a flow diagram	
14	*Descriptive data*	**(a)** Give characteristics of study participants (eg demographic, clinical, social) and information on exposures and potential confounders	144-147
		**(b)** Indicate number of participants with missing data for each variable of interest	
15	*Outcome data*	Report numbers of outcome events or summary measures	144-147

16	*Main results*	**(a)** Give unadjusted estimates and, if applicable, confounder-adjusted estimates and their precision (eg, 95% confidence interval). Make clear which confounders were adjusted for and why they were included	144-147
		**(b)** Report category boundaries when continuous variables were categorized	
		**(c)** If relevant, consider translating estimates of relative risk into absolute risk for a meaningful time period	
17	*Other analyses*	Report other analyses done—eg analyses of subgroups and interactions, and sensitivity analyses	
	**Discussion**		
18	*Key results*	Summarise key results with reference to study objectives	145-146
19	*Limitations*	Discuss limitations of the study, taking into account sources of potential bias or imprecision. Discuss both direction and magnitude of any potential bias	
20	*Interpretation*	Give a cautious overall interpretation of results considering objectives, limitations, multiplicity of analyses, results from similar studies, and other relevant evidence	146-147
21	*Generalisability*	Discuss the generalisability (external validity) of the study results	147
	**Other information**		
22	Funding	Give the source of funding and the role of the funders for the present study and, if applicable, for the original study on which the present article is based	
**TOT.**			15

STROBE CHECKLIST *CROSS-SECTIONAL STUDIES* - ARTICLE 16

TITLE: The relation between oral impacts on daily performances and perceived clinical oral conditions in primary school children in the Ugu District, Kwazulu Natal, South Africa.

Naidoo et al. (2013)

Item		Recommendation	Page n°
1	**Title and Abstract**	**(a)** Indicate the study's design with a commonly used term in the title or the abstract	214
		**(b)** Provide in the abstract an informative and balanced summary of what was done and what was found	214
	**Introduction**		
2	*Background/Rationale*	Explain the scientific background and rationale for the investigation being reported	214-215
3	*Objectives*	State specific objectives, including any prespecified hypotheses	215
	**Methods**		
4	*Study design*	Present key elements of study design early in the paper	215
5	*Setting*	Describe the setting, locations, and relevant dates, including periods of recruitment, exposure, follow-up, and data collection	215
6	*Participants*	**(a)** Give the eligibility criteria, and the sources and methods of selection of participants	215
7	*Variables*	Clearly define all outcomes, exposures, predictors, potential confounders, and effect modifiers. Give diagnostic criteria, if applicable	215

8	*Data sources/ measurement*	For each variable of interest, give sources of data and details of methods of assessment (measurement). Describe comparability of assessment methods if there is more than one group	215
9	*Bias*	Describe any efforts to address potential sources of bias	
10	*Study size*	Explain how the study size was arrived at	
11	*Quantitative variables*	Explain how quantitative variables were handled in the analyses. If applicable, describe which groupings were chosen and why	
12	*Statistical methods*	**(a)** Describe all statistical methods, including those used to control for confounding	215
		**(b)** Describe any methods used to examine subgroups and interactions	
		**(c)** Explain how missing data were addressed	
		**(d)** If applicable, describe analytical methods taking account of sampling strategy	
		**(e)** Describe any sensitivity analyses	
	**Results**		
13	*Participants*	**(a)** Report numbers of individuals at each stage of study—eg numbers potentially eligible, examined for eligibility, confirmed eligible, included in the study, completing follow-up, and analysed	215
		**(b)** Give reasons for non-participation at each stage	
		**(c)** Consider use of a flow diagram	
14	*Descriptive data*	**(a)** Give characteristics of study participants (eg demographic, clinical, social) and information on exposures and potential confounders	215-217
		**(b)** Indicate number of participants with missing data for each variable of interest	
15	*Outcome data*	Report numbers of outcome events or summary measures	215-217
16	*Main results*	**(a)** Give unadjusted estimates and, if applicable, confounder-adjusted estimates and their precision (eg, 95% confidence interval). Make clear which confounders were adjusted for and why they were included	215-217
		**(b)** Report category boundaries when continuous variables were categorized	
		**(c)** If relevant, consider translating estimates of relative risk into absolute risk for a meaningful time period	
17	*Other analyses*	Report other analyses done—eg analyses of subgroups and interactions, and sensitivity analyses	
	**Discussion**		
18	*Key results*	Summarise key results with reference to study objectives	216-217
19	*Limitations*	Discuss limitations of the study, taking into account sources of potential bias or imprecision. Discuss both direction and magnitude of any potential bias	217
20	*Interpretation*	Give a cautious overall interpretation of results considering objectives, limitations, multiplicity of analyses, results from similar studies, and other relevant evidence	216-217
21	*Generalisability*	Discuss the generalisability (external validity) of the study results	217
	**Other information**		
22	Funding	Give the source of funding and the role of the funders for the present study and, if applicable, for the original study on which the present article is based	217
**TOT.**			18

STROBE CHECKLIST *CROSS-SECTIONAL STUDIES* - ARTICLE 17

TITLE: Oral health-related quality of life among 11-12year old indigenous children in Malaysia

Nordin et al. (2019)

Item		Recommendation	Page n°
1	**Title and Abstract**	**(a)** Indicate the study's design with a commonly used term in the title or the abstract	1
		**(b)** Provide in the abstract an informative and balanced summary of what was done and what was found	1
	**Introduction**		
2	*Background/Rationale*	Explain the scientific background and rationale for the investigation being reported	2
3	*Objectives*	State specific objectives, including any prespecified hypotheses	2
	**Methods**		
4	*Study design*	Present key elements of study design early in the paper	2
5	*Setting*	Describe the setting, locations, and relevant dates, including periods of recruitment, exposure, follow-up, and data collection	2
6	*Participants*	**(a)** Give the eligibility criteria, and the sources and methods of selection of participants	2
7	*Variables*	Clearly define all outcomes, exposures, predictors, potential confounders, and effect modifiers. Give diagnostic criteria, if applicable	2-3
8	*Data sources/ measurement*	For each variable of interest, give sources of data and details of methods of assessment (measurement). Describe comparability of assessment methods if there is more than one group	
9	*Bias*	Describe any efforts to address potential sources of bias	
10	*Study size*	Explain how the study size was arrived at	2
11	*Quantitative variables*	Explain how quantitative variables were handled in the analyses. If applicable, describe which groupings were chosen and why	
12	*Statistical methods*	**(a)** Describe all statistical methods, including those used to control for confounding	3-4
		**(b)** Describe any methods used to examine subgroups and interactions	
		**(c)** Explain how missing data were addressed	
		**(d)** If applicable, describe analytical methods taking account of sampling strategy	
		**(e)** Describe any sensitivity analyses	
	**Results**		
13	*Participants*	**(a)** Report numbers of individuals at each stage of study—eg numbers potentially eligible, examined for eligibility, confirmed eligible, included in the study, completing follow-up, and analysed	4
		**(b)** Give reasons for non-participation at each stage	4
		**(c)** Consider use of a flow diagram	
14	*Descriptive data*	**(a)** Give characteristics of study participants (eg demographic, clinical, social) and information on exposures and potential confounders	4-7
		**(b)** Indicate number of participants with missing data for each variable of interest	
15	*Outcome data*	Report numbers of outcome events or summary measures	4-7

**16**	*Main results*	**(a)** Give unadjusted estimates and, if applicable, confounder-adjusted estimates and their precision (eg, 95% confidence interval). Make clear which confounders were adjusted for and why they were included	
		**(b)** Report category boundaries when continuous variables were categorized	
		**(c)** If relevant, consider translating estimates of relative risk into absolute risk for a meaningful time period	
**17**	*Other analyses*	Report other analyses done—eg analyses of subgroups and interactions, and sensitivity analyses	
	**Discussion**		
**18**	*Key results*	Summarise key results with reference to study objectives	6
**19**	*Limitations*	Discuss limitations of the study, taking into account sources of potential bias or imprecision. Discuss both direction and magnitude of any potential bias	8
**20**	*Interpretation*	Give a cautious overall interpretation of results considering objectives, limitations, multiplicity of analyses, results from similar studies, and other relevant evidence	6-8
**21**	*Generalisability*	Discuss the generalisability (external validity) of the study results	8
	**Other information**		
**22**	Funding	Give the source of funding and the role of the funders for the present study and, if applicable, for the original study on which the present article is based	9
**TOT.**			17

STROBE CHECKLIST ***CROSS-SECTIONAL STUDIES*** **- ARTICLE 18**

TITLE: Impact of oral conditions on the quality of life in schoolchildren in San Juan de Miraflores. Lima, Perú1

Paredes Martínez et al. (2014)

**Item**		**Recommendation**	**Page n°**
**1**	**Title and Abstract**	**(a)** Indicate the study's design with a commonly used term in the title or the abstract	171
		**(b)** Provide in the abstract an informative and balanced summary of what was done and what was found	171-172
	**Introduction**		
**2**	*Background/Rationale*	Explain the scientific background and rationale for the investigation being reported	172
**3**	*Objectives*	State specific objectives, including any prespecified hypotheses	172
	**Methods**		
**4**	*Study design*	Present key elements of study design early in the paper	172
**5**	*Setting*	Describe the setting, locations, and relevant dates, including periods of recruitment, exposure, follow-up, and data collection	172
**6**	*Participants*	**(a)** Give the eligibility criteria, and the sources and methods of selection of participants	172
**7**	*Variables*	Clearly define all outcomes, exposures, predictors, potential confounders, and effect modifiers. Give diagnostic criteria, if applicable	172-173

**8**	*Data sources/ measurement*	For each variable of interest, give sources of data and details of methods of assessment (measurement). Describe comparability of assessment methods if there is more than one group	172-173
**9**	*Bias*	Describe any efforts to address potential sources of bias	
**10**	*Study size*	Explain how the study size was arrived at	172
**11**	*Quantitative variables*	Explain how quantitative variables were handled in the analyses. If applicable, describe which groupings were chosen and why	
**12**	*Statistical methods*	**(a)** Describe all statistical methods, including those used to control for confounding	173
		**(b)** Describe any methods used to examine subgroups and interactions	
		**(c)** Explain how missing data were addressed	
		**(d)** If applicable, describe analytical methods taking account of sampling strategy	
		**(e)** Describe any sensitivity analyses	
	**Results**		
**13**	*Participants*	**(a)** Report numbers of individuals at each stage of study—eg numbers potentially eligible, examined for eligibility, confirmed eligible, included in the study, completing follow-up, and analysed	173
		**(b)** Give reasons for non-participation at each stage	
		**(c)** Consider use of a flow diagram	
**14**	*Descriptive data*	**(a)** Give characteristics of study participants (eg demographic, clinical, social) and information on exposures and potential confounders	173-175
		**(b)** Indicate number of participants with missing data for each variable of interest	
**15**	*Outcome data*	Report numbers of outcome events or summary measures	173-175
**16**	*Main results*	**(a)** Give unadjusted estimates and, if applicable, confounder-adjusted estimates and their precision (eg, 95% confidence interval). Make clear which confounders were adjusted for and why they were included	
		**(b)** Report category boundaries when continuous variables were categorized	
		**(c)** If relevant, consider translating estimates of relative risk into absolute risk for a meaningful time period	
**17**	*Other analyses*	Report other analyses done—eg analyses of subgroups and interactions, and sensitivity analyses	
	**Discussion**		
**18**	*Key results*	Summarise key results with reference to study objectives	175
**19**	*Limitations*	Discuss limitations of the study, taking into account sources of potential bias or imprecision. Discuss both direction and magnitude of any potential bias	
**20**	*Interpretation*	Give a cautious overall interpretation of results considering objectives, limitations, multiplicity of analyses, results from similar studies, and other relevant evidence	175-176
**21**	*Generalisability*	Discuss the generalisability (external validity) of the study results	176
	**Other information**		
**22**	Funding	Give the source of funding and the role of the funders for the present study and, if applicable, for the original study on which the present article is based	
**TOT.**			**16**

STROBE CHECKLIST *CROSS-SECTIONAL STUDIES* - ARTICLE 19

TITLE: Impact of oral diseases on daily activities among 12- To 15-year-old institutionalized orphan and non-orphan children in Bengaluru city: A cross-sectional analytical study

Pavithran et al.(2020)

Item		Recommendation	Page n°
1	**Title and Abstract**	**(a)** Indicate the study's design with a commonly used term in the title or the abstract	396
		**(b)** Provide in the abstract an informative and balanced summary of what was done and what was found	396
	**Introduction**		
2	*Background/Rationale*	Explain the scientific background and rationale for the investigation being reported	396-397
3	*Objectives*	State specific objectives, including any prespecified hypotheses	397
	**Methods**		
4	*Study design*	Present key elements of study design early in the paper	397
5	*Setting*	Describe the setting, locations, and relevant dates, including periods of recruitment, exposure, follow-up, and data collection	397
6	*Participants*	**(a)** Give the eligibility criteria, and the sources and methods of selection of participants	397
7	*Variables*	Clearly define all outcomes, exposures, predictors, potential confounders, and effect modifiers. Give diagnostic criteria, if applicable	397-398
8	*Data sources/ measurement*	For each variable of interest, give sources of data and details of methods of assessment (measurement). Describe comparability of assessment methods if there is more than one group	397-398
9	*Bias*	Describe any efforts to address potential sources of bias	
10	*Study size*	Explain how the study size was arrived at	
11	*Quantitative variables*	Explain how quantitative variables were handled in the analyses. If applicable, describe which groupings were chosen and why	
12	*Statistical methods*	**(a)** Describe all statistical methods, including those used to control for confounding	398
		**(b)** Describe any methods used to examine subgroups and interactions	
		**(c)** Explain how missing data were addressed	
		**(d)** If applicable, describe analytical methods taking account of sampling strategy	
		**(e)** Describe any sensitivity analyses	
	**Results**		
13	*Participants*	**(a)** Report numbers of individuals at each stage of study—eg numbers potentially eligible, examined for eligibility, confirmed eligible, included in the study, completing follow-up, and analysed	398
		**(b)** Give reasons for non-participation at each stage	
		**(c)** Consider use of a flow diagram	
14	*Descriptive data*	**(a)** Give characteristics of study participants (eg demographic, clinical, social) and information on exposures and potential confounders	398-400
		**(b)** Indicate number of participants with missing data for each variable of interest	
15	*Outcome data*	Report numbers of outcome events or summary measures	398-400

**16**	*Main results*	**(a)** Give unadjusted estimates and, if applicable, confounder-adjusted estimates and their precision (eg, 95% confidence interval). Make clear which confounders were adjusted for and why they were included	398-400
		**(b)** Report category boundaries when continuous variables were categorized	
		**(c)** If relevant, consider translating estimates of relative risk into absolute risk for a meaningful time period	
**17**	*Other analyses*	Report other analyses done—eg analyses of subgroups and interactions, and sensitivity analyses	
	**Discussion**		
**18**	*Key results*	Summarise key results with reference to study objectives	399
**19**	*Limitations*	Discuss limitations of the study, taking into account sources of potential bias or imprecision. Discuss both direction and magnitude of any potential bias	400-401
**20**	*Interpretation*	Give a cautious overall interpretation of results considering objectives, limitations, multiplicity of analyses, results from similar studies, and other relevant evidence	399-400
**21**	*Generalisability*	Discuss the generalisability (external validity) of the study results	401
	**Other information**		
**22**	Funding	Give the source of funding and the role of the funders for the present study and, if applicable, for the original study on which the present article is based	401
**TOT.**			18

STROBE CHECKLIST *CROSS-SECTIONAL STUDIES* - ARTICLE 20

TITLE: Quality of life related to the oral health in school from Sayausí, Cuenca Ecuador

Reinoso Vintimilla et al. (2017)

**Item**		**Recommendation**	**Page n°**
**1**	**Title and Abstract**	**(a)** Indicate the study's design with a commonly used term in the title or the abstract	227
		**(b)** Provide in the abstract an informative and balanced summary of what was done and what was found	227
	**Introduction**		
**2**	*Background/Rationale*	Explain the scientific background and rationale for the investigation being reported	228
**3**	*Objectives*	State specific objectives, including any prespecified hypotheses	228-229
	**Methods**		
**4**	*Study design*	Present key elements of study design early in the paper	229
**5**	*Setting*	Describe the setting, locations, and relevant dates, including periods of recruitment, exposure, follow-up, and data collection	229
**6**	*Participants*	**(a)** Give the eligibility criteria, and the sources and methods of selection of participants	229
**7**	*Variables*	Clearly define all outcomes, exposures, predictors, potential confounders, and effect modifiers. Give diagnostic criteria, if applicable	229

**8**	*Data sources/ measurement*	For each variable of interest, give sources of data and details of methods of assessment (measurement). Describe comparability of assessment methods if there is more than one group	229
**9**	*Bias*	Describe any efforts to address potential sources of bias	
**10**	*Study size*	Explain how the study size was arrived at	229
**11**	*Quantitative variables*	Explain how quantitative variables were handled in the analyses. If applicable, describe which groupings were chosen and why	
**12**	*Statistical methods*	**(a)** Describe all statistical methods, including those used to control for confounding	229
		**(b)** Describe any methods used to examine subgroups and interactions	
		**(c)** Explain how missing data were addressed	
		**(d)** If applicable, describe analytical methods taking account of sampling strategy	
		**(e)** Describe any sensitivity analyses	
	**Results**		
**13**	*Participants*	**(a)** Report numbers of individuals at each stage of study—eg numbers potentially eligible, examined for eligibility, confirmed eligible, included in the study, completing follow-up, and analysed	
		**(b)** Give reasons for non-participation at each stage	
		**(c)** Consider use of a flow diagram	
**14**	*Descriptive data*	**(a)** Give characteristics of study participants (eg demographic, clinical, social) and information on exposures and potential confounders	229-232
		**(b)** Indicate number of participants with missing data for each variable of interest	
**15**	*Outcome data*	Report numbers of outcome events or summary measures	229-232
**16**	*Main results*	**(a)** Give unadjusted estimates and, if applicable, confounder-adjusted estimates and their precision (eg, 95% confidence interval). Make clear which confounders were adjusted for and why they were included	229-232
		**(b)** Report category boundaries when continuous variables were categorized	
		**(c)** If relevant, consider translating estimates of relative risk into absolute risk for a meaningful time period	
**17**	*Other analyses*	Report other analyses done—eg analyses of subgroups and interactions, and sensitivity analyses	
	**Discussion**		
**18**	*Key results*	Summarise key results with reference to study objectives	232
**19**	*Limitations*	Discuss limitations of the study, taking into account sources of potential bias or imprecision. Discuss both direction and magnitude of any potential bias	
**20**	*Interpretation*	Give a cautious overall interpretation of results considering objectives, limitations, multiplicity of analyses, results from similar studies, and other relevant evidence	232-233
**21**	*Generalisability*	Discuss the generalisability (external validity) of the study results	
	**Other information**		
**22**	Funding	Give the source of funding and the role of the funders for the present study and, if applicable, for the original study on which the present article is based	
**TOT.**			15

STROBE CHECKLIST *CROSS-SECTIONAL STUDIES* - ARTICLE 21

TITLE: Oral impacts on daily performances and its socio-demographic and clinical distribution: a cross-sectional study of adolescents living in Maasai population areas, Tanzania

Simangwa et al.(2020)

Item		Recommendation	Page n°
1	**Title and Abstract**	**(a)** Indicate the study's design with a commonly used term in the title or the abstract	1
		**(b)** Provide in the abstract an informative and balanced summary of what was done and what was found	1
	**Introduction**		
2	*Background/Rationale*	Explain the scientific background and rationale for the investigation being reported	2
3	*Objectives*	State specific objectives, including any prespecified hypotheses	2
	**Methods**		
4	*Study design*	Present key elements of study design early in the paper	2
5	*Setting*	Describe the setting, locations, and relevant dates, including periods of recruitment, exposure, follow-up, and data collection	2-3
6	*Participants*	**(a)** Give the eligibility criteria, and the sources and methods of selection of participants	2-3
7	*Variables*	Clearly define all outcomes, exposures, predictors, potential confounders, and effect modifiers. Give diagnostic criteria, if applicable	3-4
8	*Data sources/ measurement*	For each variable of interest, give sources of data and details of methods of assessment (measurement). Describe comparability of assessment methods if there is more than one group	3-4
9	*Bias*	Describe any efforts to address potential sources of bias	
10	*Study size*	Explain how the study size was arrived at	3
11	*Quantitative variables*	Explain how quantitative variables were handled in the analyses. If applicable, describe which groupings were chosen and why	
12	*Statistical methods*	**(a)** Describe all statistical methods, including those used to control for confounding	4-5
		**(b)** Describe any methods used to examine subgroups and interactions	
		**(c)** Explain how missing data were addressed	
		**(d)** If applicable, describe analytical methods taking account of sampling strategy	
		**(e)** Describe any sensitivity analyses	
	**Results**		
13	*Participants*	**(a)** Report numbers of individuals at each stage of study—eg numbers potentially eligible, examined for eligibility, confirmed eligible, included in the study, completing follow-up, and analysed	5
		**(b)** Give reasons for non-participation at each stage	
		**(c)** Consider use of a flow diagram	

**14**	*Descriptive data*	**(a)** Give characteristics of study participants (eg demographic, clinical, social) and information on exposures and potential confounders	5-9
		**(b)** Indicate number of participants with missing data for each variable of interest	
**15**	*Outcome data*	Report numbers of outcome events or summary measures	5-9
**16**	*Main results*	**(a)** Give unadjusted estimates and, if applicable, confounder-adjusted estimates and their precision (eg, 95% confidence interval). Make clear which confounders were adjusted for and why they were included	
		**(b)** Report category boundaries when continuous variables were categorized	
		**(c)** If relevant, consider translating estimates of relative risk into absolute risk for a meaningful time period	
**17**	*Other analyses*	Report other analyses done—eg analyses of subgroups and interactions, and sensitivity analyses	
	**Discussion**		
**18**	*Key results*	Summarise key results with reference to study objectives	5
**19**	*Limitations*	Discuss limitations of the study, taking into account sources of potential bias or imprecision. Discuss both direction and magnitude of any potential bias	8-9
**20**	*Interpretation*	Give a cautious overall interpretation of results considering objectives, limitations, multiplicity of analyses, results from similar studies, and other relevant evidence	5-8
**21**	*Generalisability*	Discuss the generalisability (external validity) of the study results	8
	**Other information**		
**22**	Funding	Give the source of funding and the role of the funders for the present study and, if applicable, for the original study on which the present article is based	10
**TOT.**			**18**

STROBE CHECKLIST *CROSS-SECTIONAL STUDIES* - ARTICLE 22

TITLE: Impact of caries experience on quality of life related to bucal health, Machángara, Ecuador

Vélez-Vásquez et al.(2019)

**Item**		**Recommendation**	**Page n°**
**1**	**Title and Abstract**	**(a)** Indicate the study's design with a commonly used term in the title or the abstract	203
		**(b)** Provide in the abstract an informative and balanced summary of what was done and what was found	203-204
	**Introduction**		
**2**	*Background/Rationale*	Explain the scientific background and rationale for the investigation being reported	204
**3**	*Objectives*	State specific objectives, including any prespecified hypotheses	204
	**Methods**		
**4**	*Study design*	Present key elements of study design early in the paper	205
**5**	*Setting*	Describe the setting, locations, and relevant dates, including periods of recruitment, exposure, follow-up, and data collection	205

**6**	*Participants*	**(a)** Give the eligibility criteria, and the sources and methods of selection of participants	205
**7**	*Variables*	Clearly define all outcomes, exposures, predictors, potential confounders, and effect modifiers. Give diagnostic criteria, if applicable	205
**8**	*Data sources/ measurement*	For each variable of interest, give sources of data and details of methods of assessment (measurement). Describe comparability of assessment methods if there is more than one group	205
**9**	*Bias*	Describe any efforts to address potential sources of bias	
**10**	*Study size*	Explain how the study size was arrived at	205
**11**	*Quantitative variables*	Explain how quantitative variables were handled in the analyses. If applicable, describe which groupings were chosen and why	
**12**	*Statistical methods*	**(a)** Describe all statistical methods, including those used to control for confounding	205
		**(b)** Describe any methods used to examine subgroups and interactions	
		**(c)** Explain how missing data were addressed	
		**(d)** If applicable, describe analytical methods taking account of sampling strategy	
		**(e)** Describe any sensitivity analyses	
	**Results**		
**13**	*Participants*	**(a)** Report numbers of individuals at each stage of study—eg numbers potentially eligible, examined for eligibility, confirmed eligible, included in the study, completing follow-up, and analysed	205
		**(b)** Give reasons for non-participation at each stage	
		**(c)** Consider use of a flow diagram	
**14**	*Descriptive data*	**(a)** Give characteristics of study participants (eg demographic, clinical, social) and information on exposures and potential confounders	205-210
		**(b)** Indicate number of participants with missing data for each variable of interest	
**15**	*Outcome data*	Report numbers of outcome events or summary measures	205-210
**16**	*Main results*	**(a)** Give unadjusted estimates and, if applicable, confounder-adjusted estimates and their precision (eg, 95% confidence interval). Make clear which confounders were adjusted for and why they were included	205-210
		**(b)** Report category boundaries when continuous variables were categorized	
		**(c)** If relevant, consider translating estimates of relative risk into absolute risk for a meaningful time period	
**17**	*Other analyses*	Report other analyses done—eg analyses of subgroups and interactions, and sensitivity analyses	
	**Discussion**		
**18**	*Key results*	Summarise key results with reference to study objectives	210
**19**	*Limitations*	Discuss limitations of the study, taking into account sources of potential bias or imprecision. Discuss both direction and magnitude of any potential bias	211
**20**	*Interpretation*	Give a cautious overall interpretation of results considering objectives, limitations, multiplicity of analyses, results from similar studies, and other relevant evidence	210-211
**21**	*Generalisability*	Discuss the generalisability (external validity) of the study results	211
	**Other information**		
**22**	Funding	Give the source of funding and the role of the funders for the present study and, if applicable, for the original study on which the present article is based	211
**TOT.**			19

STROBE CHECKLIST *CROSS-SECTIONAL STUDIES* - ARTICLE 23

TITLE: Orthodontic Treatment Need, Self-Esteem, and Oral Health-Related Quality of Life Assessment of Primary Schoolchildren: A Cross-Sectional Pilot Study

Yetkiner et al.(2014)

Item		Recommendation	Page n°
1	**Title and Abstract**	**(a)** Indicate the study's design with a commonly used term in the title or the abstract	182
		**(b)** Provide in the abstract an informative and balanced summary of what was done and what was found	182
	**Introduction**		
2	*Background/Rationale*	Explain the scientific background and rationale for the investigation being reported	182-183
3	*Objectives*	State specific objectives, including any prespecified hypotheses	183
	**Methods**		
4	*Study design*	Present key elements of study design early in the paper	183
5	*Setting*	Describe the setting, locations, and relevant dates, including periods of recruitment, exposure, follow-up, and data collection	183
6	*Participants*	**(a)** Give the eligibility criteria, and the sources and methods of selection of participants	183
7	*Variables*	Clearly define all outcomes, exposures, predictors, potential confounders, and effect modifiers. Give diagnostic criteria, if applicable	183-184
8	*Data sources/ measurement*	For each variable of interest, give sources of data and details of methods of assessment (measurement). Describe comparability of assessment methods if there is more than one group	183-184
9	*Bias*	Describe any efforts to address potential sources of bias	
10	*Study size*	Explain how the study size was arrived at	183
11	*Quantitative variables*	Explain how quantitative variables were handled in the analyses. If applicable, describe which groupings were chosen and why	
12	*Statistical methods*	**(a)** Describe all statistical methods, including those used to control for confounding	184
		**(b)** Describe any methods used to examine subgroups and interactions	
		**(c)** Explain how missing data were addressed	
		**(d)** If applicable, describe analytical methods taking account of sampling strategy	
		**(e)** Describe any sensitivity analyses	
	**Results**		
13	*Participants*	**(a)** Report numbers of individuals at each stage of study—eg numbers potentially eligible, examined for eligibility, confirmed eligible, included in the study, completing follow-up, and analysed	184
		**(b)** Give reasons for non-participation at each stage	
		**(c)** Consider use of a flow diagram	
14	*Descriptive data*	**(a)** Give characteristics of study participants (eg demographic, clinical, social) and information on exposures and potential confounders	184-187

		**(b)** Indicate number of participants with missing data for each variable of interest	
**15**	*Outcome data*	Report numbers of outcome events or summary measures	184-187
**16**	*Main results*	**(a)** Give unadjusted estimates and, if applicable, confounder-adjusted estimates and their precision (eg, 95% confidence interval). Make clear which confounders were adjusted for and why they were included	184-187
		**(b)** Report category boundaries when continuous variables were categorized	
		**(c)** If relevant, consider translating estimates of relative risk into absolute risk for a meaningful time period	
**17**	*Other analyses*	Report other analyses done—eg analyses of subgroups and interactions, and sensitivity analyses	
	**Discussion**		
**18**	*Key results*	Summarise key results with reference to study objectives	185
**19**	*Limitations*	Discuss limitations of the study, taking into account sources of potential bias or imprecision. Discuss both direction and magnitude of any potential bias	185
**20**	*Interpretation*	Give a cautious overall interpretation of results considering objectives, limitations, multiplicity of analyses, results from similar studies, and other relevant evidence	185-188
**21**	*Generalisability*	Discuss the generalisability (external validity) of the study results	188
	**Other information**		
**22**	Funding	Give the source of funding and the role of the funders for the present study and, if applicable, for the original study on which the present article is based	
**TOT.**			18

Table S2. Evidence Tables.

Evidence Table for Cross Sectional Studies (Based on Flc 3.0, Designed by Authors)

This is the summary table for the evaluation of the methodological quality of the study. Keeping in mind your answers to the 6 areas that appear on this screen, assess the quality of the evidence provided by the analysed study.

Research Question<br>*Is the study based on a clearly defined research question?*	**YES**	**NO**	**PARTIALLY**	**WITHOUT DATA/INFO**
Method<br>*Has the study method allowed to minimize bias?*	**YES**	**NO**	**PARTIALLY**	**WITHOUT DATA/INFO**
Results<br>*Are the results correctly synthesized and described?*	**YES**	**NO**	**PARTIALLY**	**WITHOUT DATA/INFO**
Conclusions<br>*Are the conclusions justified?*	**YES**	**NO**	**PARTIALLY**	**WITHOUT DATA/INFO**
External validity<br>*Are study results generalizable to the population and context that interest?*	**YES**	**NO**	**PARTIALLY**	**WITHOUT DATA/INFO**
Conflict of interest<br>*Is the existence or absence of conflict of interest well described?*	**YES**	**NO**	**PARTIALLY**	**WITHOUT DATA/INFO**

As guidelines you may consider the following suggestions:

	**Method YES**	**Method PARTIALLY**	**Method NO**
**Majority other criteria YES**	High quality	Medium quality	Low quality
**Majority other criteria PARTIALLY**	Medium quality	Medium quality	Low quality
**Majority other criteria NO**	Low quality	Low quality	Low quality

REFE-RENCE **Author/year**	**RESEARCH QUESTION (population/intervention/ outcome)**	**METHODOLOGY**	**RESULTS**	**CONCLUSIONS**	**CONFLICT OF INTEREST**	**EXTERNAL VALIDITY**	QUALITY OF THE STUDY **High/medium/low**
ALVES et al. 2015	**Population:** **12 years old** <br>**Intervention:** Clinical exam; C-OIDP; family development index (FDI); interview on propensity-related need. <br>**Outcomes:** **Impact prevalence; mean C-OIDP**	**Study design:** Cross-sectional <br>**Objectives:** stated <br>**Setting and time:** - **setting**: Manguinhos - **time:** not stated <br>**Eligibility criteria:** Stated **Statistical analysis:** Association analysis with Chi square test; Kruskall Wallis test; Spearman correlation coefficient; McNemar´s test. <br>**Other:** Sample size calculation. Simple random sampling. <br>**Absent:** examiners calibration;	**Correctly synthesized and described:** Yes	**Are conclusions justified?** Partially	**Is it described? Yes**	**Are results generalisable to the general population?** <br>**No, only to deprived groups of adolescents.**	MEDIUM

	questionnaire C-OIDP not described					
YES	PARTIALLY	YES	PARTIALLY	YES	PARTIALLY	**MEDIUM**

REFE-RENCE **Author/year**	**RESEARCH QUESTION (population/intervention/ outcome)**	**METHODOLOGY**	**RESULTS**	**CONCLUSIONS**	**CONFLICT OF INTEREST**	**EXTERNAL VALIDITY**	QUALITY OF THE STUDY **High/medium/low**
ALZAHRANI et al. 2019	**Population:** **Males 12 –15 years old** <br>**Intervention:** Clinical exam; C-OIDP; questionnaire with sociodemographic data <br>**Outcomes:** **Impact prevalence; mean C-OIDP**	**Study design:** Cross-sectional <br>**Objectives:** Stated <br>**Setting and time:** - **setting**: not stated - **time:** Nov 2017 to Jan 2018 <br>**Eligibility criteria:** Stated; females not included for cultural reasons, as examiners were men. <br>**Statistical analysis:** association analysis with Chi square test; Multivariate logistic regression model; Odds ratio; Nagelkerke R2 value <br>**Other:** Sample size calculation. 2 stage randomised sampling method.	**Correctly synthesized and described:** Yes	**Are conclusions justified?** Partially <br>**Mistake in conclusions**: <severity of impacts was high>: majority of participants reported moderate or minor severity. They say being a cross sectional design is a "limitation" itself. Implications of key findings is not clear. Poor conclusions.	**Is it described?** **Yes**	**Are results generalisable to the general population?** <br>**No; only to males in that Arabic region. Sample from 3 dissimilar areas.**	MEDIUM

	Clinical exam on WHO guideline 1997. Adaptation of the C-OIDP to Arabic. Explain the C-OIDP questionnaire<br><br>**Mistake:** written "intra-examiner agreement" while they refer to "inter-examiner agreement" (between 2 different examiners.) Only males participants						
YES	PARTIALLY	YES	PARTIALLY	YES	NO	MEDIUM	

REFE-RENCE Author/year	RESEARCH QUESTION (population/intervention/ outcome)	METHODOLOGY	RESULTS	CONCLUSIONS	CONFLICT OF INTEREST	EXTERNAL VALIDITY	QUALITY OF THE STUDY High/medium/low
AMALIA et al. 2015	**Population:** **12 years old**<br><br>**Intervention:** Clinical exam; CS-C-OIDP; questionnaire with sociodemographic data<br><br>**Outcomes:** **Impact prevalence; mean CS-C-OIDP**	**Study design:** Cross-sectional<br><br>**Objectives:** Stated<br><br>**Setting and time:** not stated<br><br>**Eligibility criteria:** Stated<br><br>**Statistical analysis:** association analysis Chi square test; Regression analysis; negative binomial regression analysis<br><br>**Other:** Sample size calculation: not based on power analysis. 4 dental nurses were trained to collect C-OIDP. Good explanation of the survey.	**Correctly synthesized and described:** Yes	**Are conclusions justified?** Partially Validity of CS-C-OIDP as an outcome measure for oral health programs has not been yet established. A more detailed measurements of caries is required than DT used in this study. Poor conclusions.	**Is it described? Yes**	**Are results generalisable to the general population? No. (convenience sample, sample size not based on power analysis)**	MEDIUM

	Inter-examiner and intra-examiner reliability were assessed.<br><br>**Limitation:** Convenience sample					
YES	PARTIALLY	YES	PARTIALLY	YES	NO	**MEDIUM**

REFE-RENCE Author/year	RESEARCH QUESTION (population/intervention/ outcome)	METHODOLOGY	RESULTS	CONCLUSIONS	CONFLICT OF INTEREST	EXTERNAL VALIDITY	QUALITY OF THE STUDY High/medium/low
ATHIRA et al. 2015	**Population:** **12 -17 years old** **Intervention:** Clinical exam; C-OIDP; questionnaire with sociodemographic data **Outcomes:** **missing Impact prevalence; mean C-OIDP categorised**	**Study design:** Cross-sectional **Objectives:** Stated **Setting and time:** - **setting**: stated - **time**: not stated **Eligibility criteria:** Stated **Statistical analysis:** association analysis with Chi square test; Student´s t test; ANOVA. **Other:** Sample size calculation based on power analysis. C-OIDP: good explanation of the survey. Intraexaminer reliability was assessed.	**Correctly synthesized and described:** missing mean C-OIDP score. Otherwise, OK.	**Are conclusions justified?** Partially. "Participants were in early stage of adolescence and hence no gender difference was found in C-OIDP" (not exactly, age spam was: 12-17 years old, that is not "early stage"). 37,3% had C-OIDP score between 1-10 ("considerable impact" in the opinion of the author, we disagree). "The majority of subjects scored zero C-OIDP" (wrong: only 43,1%). Poor conclusions.	**Is it described?** **No**	**Are results generalisable to the general population?** **No (convenience sample)**	LOW

	**Limitation:** Convenience sample					
YES	PARTIALLY	YES	PARTIALLY	NO	NO	**LOW**

REFE-RENCE Author/year	RESEARCH QUESTION (population/intervention/ outcome)	METHODOLOGY	RESULTS	CONCLUSIONS	CONFLICT OF INTEREST	EXTERNAL VALIDITY	QUALITY OF THE STUDY High/medium/low
BAKTIAR et al. 2014	**Population:** **11-13 years old** **Intervention:** **C**linical exam; questionnaire **Outcomes:** **Impact prevalence; Mean C-OIDP score** DMFT; Loe and Silnes plaque index	**Study design:** Cross sectional **Objectives:** Stated **Setting and time:** - **setting**: stated - **time:** time period 2012 **Eligibility criteria:** inclusion criteria not stated; exclusion criteria stated **Statistical analysis:** Random cluster sample; Kolmogorov Smirnov test; non parametric Mann Whitney U-test and Spearman correlation. **Mistake:** Some mistakes in English writing	**Correctly synthesized and described:** No Mean C-OIDP score shows different value in "Results" and "Material and methods" **In "Results":** There is association between DMFT/C-OIDP and bacterial plaque/ C-OIDP. **In "Discussion":** There is also association between malocclusion/ C-OIDP.	**Are conclusions justified?** Partially	**Is it described?** **No**	**Are results generalisable to the general population?** **Partially**	MEDIUM

YES	PARTIALLY	PARTIALLY	PARTIALLY	NO	PARTIALLY	**MEDIUM**

REFE-RENCE Author/year	RESEARCH QUESTION (population/intervention/ outcome)	METHODOLOGY	RESULTS	CONCLUSIONS	CONFLICT OF INTEREST	EXTERNAL VALIDITY	QUALITY OF THE STUDY High/medium/low
BASAVARAJ et al. 2014	**Population:** **12 and 15 years old**<br><br>**Intervention:** Clinical exam; C-OIDP; questionnaire with sociodemographic data<br><br>**Outcomes:** **Impact prevalence; mean C-OIDP**	**Study design:** Cross-sectional<br><br>**Objectives:** Stated<br><br>**Setting and time:**<br>- **setting**: stated<br>- **time:** August-November 2012<br><br>**Eligibility criteria:** exclusion stated<br><br>**Statistical analysis:** descriptive statistics; bivariate analysis with Chi square test; multiple logistic regression analysis<br><br>**Other:** Sample size calculation: based on power analysis. C-OIDP: good explanation of the survey. Intra-examiner	**Correctly synthesized and described:** Yes	**Are conclusions justified?** Yes	**Is it described?** **Yes**	**Are results generalisable to the general population? Yes, a city of approx. 180.000 hab.**	HIGH

	reliability was assessed.; also, inter-examiner reliability. Examiner was calibrated. 2 staged cluster sampling technique. Schools and subjects randomly selected.					
YES	YES	YES	YES	YES	YES	**HIGH**

REFE-RENCE **Author/year**	**RESEARCH QUESTION (population/intervention/ outcome)**	**METHODOLOGY**	**RESULTS**	**CONCLUSIONS**	**CONFLICT OF INTEREST**	**EXTERNAL VALIDITY**	QUALITY OF THE STUDY **High/medium/low**
BERNABE' et al. 2007	**Population:** **11-12 years old** **Intervention:** Questionnaire **Outcomes:** **Impact prevalence; Mean C-OIDP score**	**Study design:** Not stated although it is cross-sectional **Objectives:** Stated **Setting and time:** - **setting**: stated - **time:** time period 2006 **Eligibility criteria:** Stated only inclusion criteria **Statistical analysis**: Bivariate analysis with chi square test, Mann-Whitney test and Kruskal Wallis test **Other:** Random cluster sampling; Sample size calculation based on impact prevalence of	**Correctly synthesized and described?** Yes	**Are conclusions justified?** Yes	**Is it described? No**	**Are results generalisable to the general population? No, only to low-income urban communities in Peru.**	MEDIUM

	50%, error of 5% and design effect of 2,5. Ethical approval obtained.					
YES	PARTIALLY	YES	YES	NO	NO	**MEDIUM**

REFE-RENCE Author/year	RESEARCH QUESTION (population/intervention/ outcome)	METHODOLOGY	RESULTS	CONCLUSIONS	CONFLICT OF INTEREST	EXTERNAL VALIDITY	QUALITY OF THE STUDY High/medium/low
BIANCO et al. 2009	**Population:** **11-16 years old**<br><br>**Intervention:** **Questionnaire; Interview; Oral examination**<br><br>**Outcomes:** **Impact prevalence; Mean C-OIDP score**	**Study design:** Cross sectional<br><br>**Objectives:** Stated<br><br>**Setting and time:**<br>- **setting**: stated<br>- **time:** January to April 2006<br><br>**Eligibility criteria:** Not stated<br><br>**Statistical analysis:** Stepwise multiple linear and logistic regression models<br><br>**Other:** Randomly selected schools and participants. Pilot study. Informed consent obtained. Usage of pathologies list. Cross cultural	Correctly synthesized and described? Yes	Are conclusions justified? Yes	**Is it described? No**	**Are results generalisable to the general population? Partially (although sample selection was randomised, the selection method is not explained)**	HIGH

	translation and validation to Italian. Ethics Committee approval.					
YES	YES	YES	YES	NO	PARTIALLY	**MEDIUM**

REFE-RENCE Author/year	RESEARCH QUESTION (population/intervention/ outcome)	METHODOLOGY	RESULTS	CONCLUSIONS	CONFLICT OF INTEREST	EXTERNAL VALIDITY	QUALITY OF THE STUDY High/medium/low
CASTRO et al. 2011	**Population:** **11-12 years old** **Intervention:** Questionnaire; clinical exam; interview **Outcomes:** **Impact prevalence; mean C-OIDP score**	**Study design:** Cross-sectional **Objectives:** Stated **Setting and time:** - **setting**: stated - **time:** November 2007 and July 2008 **Eligibility criteria:** Informed consent returned **Statistical analysis:** Bivariate analysis with Mann Whitney test; logistic regression analysis **Other:** Probabilistic sample with complex design. Usage of pathologies list. Calibrated examiners. Clinical exam was visual without a probe. Children were randomly	**Correctly synthesized and described?** Yes	**Are conclusions justified?** Yes	**Is it described?** **No**	**Are results generalisable to the general population?** **No**	MEDIUM

		selected from the subset of consent forms returned.						
YES		PARTIALLY	YES	YES	NO	NO	MEDIUM	

REFE-RENCE Author/year	RESEARCH QUESTION (population/intervention/ outcome)	METHODOLOGY	RESULTS	CONCLUSIONS	CONFLICT OF INTEREST	EXTERNAL VALIDITY	QUALITY OF THE STUDY High/medium/low
DEL CASTILLO LOPEZ et al. 2014	**Population:** **11-12 years old** **Intervention:** **Questionnaire** **Outcome:** **Impact prevalence; Mean C-OIDP**	**Study design:** Cross sectional **Objectives:** Stated **Setting and time:** - **setting**: stated - **time:** Time not specified, only year **Eligibility criteria:** Stated **Statistical analysis:** Not stated statistical test used **Other:** Some references quoted in the text do not appear in "References". **Sample selection:** convenience	Correctly synthesized and described: Yes	Are conclusions justified? Yes	**Is it described? No**	**Are results generalisable to the general population? No, it's a convenience sample**	MEDIUM

.						
YES	PARTIALLY	YES	YES	NO	NO	**MEDIUM**

REFE-RENCE **Author/year**	**RESEARCH QUESTION (population/intervention/ outcome)**	**METHODOLOGY**	**RESULTS**	**CONCLUSIONS**	**CONFLICT OF INTEREST**	**EXTERNAL VALIDITY**	QUALITY OF THE STUDY **High/medium/low**
DO et al. 2020	**Population:** **12 and 15 years old** **Intervention:** **Questionnaire** **Outcomes:** **Impact prevalence; Mean C-OIDP score**	**Study design:** Cross sectional **Objectives:** Stated **Setting and time:** - **setting**: stated - **time:** 2011 **Eligibility criteria:** Stated **Statistical analysis:** Chi square test, Fisher test, Mann Whitney test **Others:** Examiner's calibration not done	**Correctly synthesized and described?** Yes	**Are conclusions justified?** Yes	**Is it described?** **Yes**	**Are results generalisable to the general population?** **Yes**	HIGH
	YES	YES	YES	YES	YES	YES	HIGH

REFERENCE **Author/year**	**RESEARCH QUESTION (population/intervention/ outcome)**	**METHODOLOGY**	**RESULTS**	**CONCLUSIONS**	**CONFLICT OF INTEREST**	**EXTERNAL VALIDITY**	QUALITY OF THE STUDY **High/medium/low**
DUMITRACHE et al. 2009	**Population:** **11-13 years old** **Intervention:** **Questionnaire** **Outcomes:** **Impact prevalence**	**Study design:** Cross sectional **Objectives:** Stated **Setting and time:** - **setting**: stated - **time:** not stated **Eligibility criteria:** Not stated **Statistical analysis:** statistical tests not described **Others:** Several mistakes in English text; random selection.	**Correctly synthesized and described:** Yes	**Are conclusions justified?** Partially	**Is it described?** **No**	**Are results generalisable to the general population?** **No, it's a convenience simple**	LOW
	PARTIALLY	NO	YES	PARTIALLY	NO	NO	LOW

REFE-RENCE<br>**Author/year**	**RESEARCH QUESTION (population/intervention/ outcome)**	**METHODOLOGY**	**RESULTS**	**CONCLUSIONS**	**CONFLICT OF INTEREST**	**EXTERNAL VALIDITY**	QUALITY OF THE STUDY<br>**High/medium/low**
KUMAR et al.<br>2015	**Population:**<br>**12 - 15 years old**<br><br>**Intervention:**<br>Clinical exam; C-OIDP; questionnaire with sociodemographic and oral health related behaviours data<br><br>**Outcomes:**<br>**Impact prevalence; mean C-OIDP by performance (not overall mean score)**	**Study design:** Cross-sectional<br><br>**Objectives:** Stated<br><br>**Setting and time:**<br>- **setting**: stated<br>- **time:** June and July 2014<br><br>**Eligibility criteria:** Stated<br><br>**Statistical analysis:** ANOVA, t-test; bivariate analysis; multiple logistic regression analysis<br><br>**Other:** Sample size calculation: based on prevalence of dental caries; no more info. Examiner was calibrated. 2 staged cluster sampling technique.	Correctly synthesized and described? Yes	Are conclusions justified? Yes	**Is it described? No**	**Are results generalisable to the general population? Yes**	MEDIUM

		Schools were randomly selected					
	YES	PARTIALLY	YES	YES	NO	YES	MEDIUM

REFE-RENCE **Author/year**	**RESEARCH QUESTION (population/intervention/ outcome)**	**METHODOLOGY**	**RESULTS**	**CONCLUSIONS**	**CONFLICT OF INTEREST**	**EXTERNAL VALIDITY**	QUALITY OF THE STUDY **High/medium/low**
MARCELO et al. 2015	**Population:** **11 and 12 years old**<br><br>**Intervention:** **Q**uestionnaire<br><br>**Outcome:** Mean C-OIDP score; Impact prevalence	**Study design:** Cross sectional<br><br>**Objectives:** Stated<br><br>**Setting and time:**<br>- **setting**: stated<br>- **time:** August and September 2013<br><br>**Eligibility criteria:** inclusion criteria stated<br><br>**Statistical analysis:**	**Correctly synthesized and described?** Yes	**Are conclusions justified?** Yes	**Is it described?** **No**	**Are results generalisable to the general population? No. Not homogeneous sample, only internal validity.**	HIGH

		Chi square test and Mann Whitney U test<br><br>**Other:**<br>Some misprint wording in Spanish text					
	YES	YES	YES	YES	NO	NO	HIGH

REFE-RENCE<br>**Author/year**	**RESEARCH QUESTION (population/intervention/ outcome)**	**METHODOLOGY**	**RESULTS**	**CONCLUSIONS**	**CONFLICT OF INTEREST**	**EXTERNAL VALIDITY**	QUALITY OF THE STUDY<br>**High/medium/low**
MORENO et al. 2014	**Population:**<br>**11 - 14 years old**<br><br>**Intervention:**<br>**Q**uestionnaire<br><br>**Outcomes:**<br>Mean C-OIDP score; Impact prevalence	**Study design:** Cross sectional<br><br>**Objectives:** Stated<br><br>**Setting and time:**<br>- **setting**: stated<br>- **time:** September 2013	**Correctly synthesized and described?**<br>Yes	**Are conclusions justified?**<br>Partially	**Is it described?**<br>**Yes**	**Are results generalisable to the general population?**<br>**No**	MEDIUM

		**Eligibility criteria:** Not stated<br><br>**Statistical analysis:** Not-parametric tests; Chi square test, Mann Whitney U test and Kruskal Wallis<br><br>**Other:** Do not mention study limitations, sample selection method, sample size calculation					
	YES	PARTIALLY	YES	PARTIALLY	YES	NO	MEDIUM

REFE-RENCE **Author/year**	**RESEARCH QUESTION (population/intervention/ outcome)**	**METHODOLOGY**	**RESULTS**	**CONCLUSIONS**	**CONFLICT OF INTEREST**	**EXTERNAL VALIDITY**	QUALITY OF THE STUDY **High/medium/low**
NAIDOO et al. 2013	**Population:** **11-13 years old**<br><br>**Intervention:** Questionnaire<br><br>**Outcomes:** Impact prevalence	**Study design:** Cross sectional<br><br>**Objectives:** Stated<br><br>**Setting and time:**<br>- **setting**: stated<br>- **time:** not stated	**Correctly synthesized and described?** Yes	**Are conclusions justified?** Yes	**Is it described? Yes**	**Are results generalisable to the general population? No. Sample belongs to low socioeconomical level, it is not representative of the entire**	MEDIUM

	**Eligibility criteria:** Not stated<br><br>**Statistical analysis:** Chi square test and Mann Whitney U test. Only bivariate analysis and not multivariate<br><br>**Other:** Random selection of schools participating. 3 examiners were calibrated. Intra and inter-examiner reliability was assessed. They compare index outcome obtained, with studies done in adults			**Y**	**country (South Africa).**	
PARTIALLY	PARTIALLY	YES	YES	YES	NO	MEDIUM

REFE-RENCE Author/year	RESEARCH QUESTION (population/intervention/ outcome)	METHODOLOGY	RESULTS	CONCLUSIONS	CONFLICT OF INTEREST	EXTERNAL VALIDITY	QUALITY OF THE STUDY High/medium/low
NORDIN et al. 2019	**Population:** **11-12 years old**<br><br>**Intervention:** **Questionnaire**<br><br>**Outcome:** Impact prevalence; Mean C-OIDP score	**Study design:** Cross sectional<br><br>**Objectives:** Stated<br><br>**Setting and time:** - **setting**: stated - **time:** November 2014<br><br>**Eligibility criteria:** Stated<br><br>**Statistical analysis:** Distribution of C-OIDP scores was skewed, therefore non parametric tests were used (Kruskal Wallis and Mann Whitney). Also, multiple logistic regression.<br><br>**Other:** Sample size determination based on previous impact	**Correctly synthesized and described?** Yes	**Are conclusions justified?** Partially	**Is it described? Yes**	**Are results generalisable to the general population? No, only to indigenous rural population where the sample comes from.**	HIGH

		prevalence. Pilot study. Examiners calibrated.					
	YES	YES	YES	PARTIALLY	YES	NO	HIGH

REFE-RENCE **Author/year**	**RESEARCH QUESTION (population/intervention/ outcome)**	**METHODOLOGY**	**RESULTS**	**CONCLUSIONS**	**CONFLICT OF INTEREST**	**EXTERNAL VALIDITY**	QUALITY OF THE STUDY **High/medium/low**
PAREDES et al. 2014	**Population:** **11-12 years old** **Intervention:** **Questionnaire** **Outcomes:** Impact prevalence	Study design: Cross sectional Objectives: Stated Setting and time: - **setting:** not stated - **time:** 2013	Correctly synthesized and described? Yes	Are conclusions justified? Partially	**Is it described? No**	**Are results generalisable to the general population? Partially; they do not explain representativeness of the sample neither sample selection process.**	MEDIUM

		**Eligibility criteria:** Stated<br><br>**Statistical analysis:** “sample was obtained statistically”, they do not explain how it was obtained<br><br>**Other:** Pilot study was done					
	PARTIALLY	PARTIALLY	YES	PARTIALLY	NO	PARTIALLY	MEDIUM

REFE-RENCE **Author/year**	**RESEARCH QUESTION (population/intervention/ outcome)**	**METHODOLOGY**	**RESULTS**	**CONCLUSIONS**	**CONFLICT OF INTEREST**	**EXTERNAL VALIDITY**	QUALITY OF THE STUDY **High/medium/low**
PAVITRAN et al. 2020	**Population:** **12-15 years old**<br><br>**Intervention:** demographic questionnaire; C-OIDP questionnaire	**Study design:** Cross sectional analytical study<br><br>**Objectives:** Not stated	**Correctly synthesized and described?** Yes	**Are conclusions justified?** Yes	**Is it described? Yes**	**Are results generalisable to the general population? Yes. Author states good**	HIGH

	**Outcomes: Impact prevalence; Mean C-OIDP score;** Describes null and alternative hypothesis	**Setting and time:** - **setting**: not stated - **time:** March to July 2014<br><br>**Eligibility criteria**: Stated<br><br>**Statistical analysis:** descriptive statistics; association analysis: Chi-square test, Mann Whitney U test (comparison of mean C-OIDP score between 2 groups); bivariate association with ANOVA. One-way analysis of variance, Step-wise multiple linear regression<br><br>**Other:** Ethical Committee clearance with specific number. Study presents information based on Strobe statement 2007 checklist. Examiner trained and calibrated, intra-examiner		**external validity**	

		reliability assessed. Pilot study done. Formula for calculation of sample size. Simple random sampling technique for choosing orphanages and convenience selection for non orphanages participants. Oral health education program was conducted after examination					
	YES	YES	YES	YES	YES	YES	HIGH

REFE-RENCE **Author/year**	**RESEARCH QUESTION (population/intervention/ outcome)**	**METHODOLOGY**	**RESULTS**	**CONCLUSIONS**	**CONFLICT OF INTEREST**	**EXTERNAL VALIDITY**	QUALITY OF THE STUDY **High/medium/low**
REINOSO et al. 2017	**Population:** **11-12 years old** **Intervention: Questionnaire** **Outcome:** Impact prevalence	**Study design:** Cross sectional **Objectives:** Stated **Setting and time:** - **setting**: stated - **time:** May to July 2015	**Correctly synthesized and described?** Yes	**Are conclusions justified?** Yes	**Is it described? No**	**Are results generalisable to the general population? Partially, only generalizable to study population.**	MEDIUM

		**Eligibility criteria:** Stated<br><br>**Statistical analysis:** Impact extent (U de Mann Whitney); Impact intensity (Chi square test and Fisher exact test).<br><br>**Other:** Examiner calibration; Pilot study; Do not explain sample selection technique.					
	YES	PARTIALLY	YES	YES	NO	PARTIALLY	**MEDIUM**

REFE-RENCE **Author/year**	**RESEARCH QUESTION (population/intervention/ outcome)**	**METHODOLOGY**	**RESULTS**	**CONCLUSIONS**	**CONFLICT OF INTEREST**	**EXTERNAL VALIDITY**	QUALITY OF THE STUDY **High/medium/low**
SIMANGWA et al. 2020	**Population: 12-17 years old**<br><br>**Intervention:** clinical exam; questionnaire; Kiswahili version of C-OIDP	**Study design:** Cross sectional<br><br>**Objectives:** Stated<br><br>**Setting and time:**<br>- **setting:** stated<br>- **time:** June to November 2016.	**Correctly synthesized and described?** Yes	**Are conclusions justified?** Yes	**Is it described? Yes**	**Are results generalisable to the general population? Partially (due to very strict inclusion criteria).**	HIGH

	**Outcomes:**<br>**Impact prevalence:** Wealth index (PCA: principal component analysis method); Simplified oral hygiene index (OHI-S); Gingival bleeding index; Dental caries assessed by WHO criteria (Basic methods 2013); Dental fluorosis assessed by TF index.	**Eligibility criteria:** Stated<br><br>**Statistical analysis:** bivariate analysis with Pearson Chi square test; Stepwise multiple variable logistic regression analysis.<br><br>**Other:** One stage cluster sample design. Sample size estimation based on prevalence of dental erosion. Examiner trained and calibrated. Inter-examiner assessment for dental erosion. Intra-examiner concordance done.					
	YES	YES	YES	YES	YES	PARTIALLY	**HIGH**

REFE-RENCE Author/year	RESEARCH QUESTION (population/intervention/ outcome)	METHODOLOGY	RESULTS	CONCLUSIONS	CONFLICT OF INTEREST	EXTERNAL VALIDITY	QUALITY OF THE STUDY High/medium/low
VELEZ et al. 2019	**Population:** **11-12 years old**<br><br>**Intervention:** **Questionnaire**<br><br>**Outcomes:** Impact prevalence	**Study design:** Cross sectional<br><br>**Objectives:** Stated<br><br>**Setting and time:**<br>- **setting**: stated<br>- **time:** not stated<br><br>**Eligibility criteria:** Stated<br><br>**Statistical analysis:** Chi square test; Pearson R; simple correspondence analysis.<br><br>**Other:** Sample selection randomised, stratified and proportional. Examiner calibration.	**Correctly synthesized and described?** Yes	**Are conclusions justified?** Yes	**Is it described?** **Yes**	**Are results generalisable to the general population? Partially; it is not clear if they did stratification based on parents socioeconomical level.**	HIGH
	YES	YES	YES	YES	YES	PARTIALLY	HIGH

REFE-RENCE Author/year	RESEARCH QUESTION (population/intervention/ outcome)	METHODOLOGY	RESULTS	CONCLUSIONS	CONFLICT OF INTEREST	EXTERNAL VALIDITY	QUALITY OF THE STUDY High/medium/low
YETKINER et al. 2014	**Population:** **13-14 years old** **Intervention:** clinical exam; questionnaire; interview **Outcomes:** **Impact prevalence; Mean C-OIDP score per dimensions; IOTN-DHC (index of orthodontic treatment need dental component). IOTN-AC (index of orthodontic treatment need aesthetic component). Rosenberg self-steem scale**	**Study design:** Cross sectional **Objectives:** Stated **Setting and time:** - **setting**: stated - **time:** not stated **Eligibility criteria:** Stated **Statistical analysis:** Spearman rho; Kruskal Wallis test; Chi-square test. **Other:** Inter-operator and intra-operator reliability tested. Sample size and selection method not stated.	**Correctly synthesized and described?** Yes	**Are conclusions justified?** Partially	**Is it described?** **No**	**Are results generalisable to the general population?** **No**	MEDIUM
	YES	PARTIALLY	YES	PARTIALLY	NO	NO	MEDIUM
